# Efficacy of preventive use of oxygen therapy after planned extubation in high-risk patients with extubation failure: A network meta-analysis of randomized controlled trials

**DOI:** 10.3389/fmed.2022.1026234

**Published:** 2022-10-13

**Authors:** Xiaozhuo Zheng, Rui Wang, Mohan Giri, Jun Duan, Mengyi Ma, Shuliang Guo

**Affiliations:** ^1^Department of Pulmonary and Critical Care Medicine, The First Affiliated Hospital of Chongqing Medical University, Chongqing, China; ^2^Department of Thoracic Surgery, The Third Affiliated Hospital of Chongqing Medical University, Chongqing, China

**Keywords:** high-flow nasal catheter, extubation failure, reintubation, respiratory failure, non-invasive ventilation, high-risk patients, network meta-analysis

## Abstract

**Background:**

Extubation failure is common in critically ill patients, especially those with high-risk factors, and is associated with poor prognosis. Prophylactic use of oxygen therapy after extubation has been gradually introduced. However, the best respiratory support method is still unclear.

**Purpose:**

This study aimed to evaluate the efficacy of four post-extubation respiratory support approaches in reducing reintubation and respiratory failure in patients at high-risk of extubation failure.

**Methods:**

A comprehensive search was performed in Cochrane Central Register of Controlled Trials, PubMed, EMBASE, and Web of Science from inception to June 2022. Randomized controlled trials (RCTs) comparing post-extubation preventive use of respiratory management strategies, including conventional oxygen therapy (COT), non-invasive ventilation (NIV), and high-flow nasal catheter (HFNC) in high-risk patients with extubation failure were reviewed. Primary outcomes were reintubation rate and respiratory failure. Secondary outcomes included intensive care unit (ICU) mortality, ICU stay and length of hospital stay (LOS).

**Results:**

Seventeen RCTs comprising 2813 participants were enrolled. Compared with COT, the three respiratory support methods (NIV, HFNC, NIV + HFNC) were all effective in preventing reintubation [odds ratio (OR) 0.46, 95% confidence interval (CI) 0.32–0.67; OR 0.26, 95% CI 0.14–0.48; OR 0.62, 95% CI 0.39–0.97, respectively] and respiratory failure (OR 0.23, 95% CI 0.10–0.52; OR 0.15, 95% CI 0.04–0.60; OR 0.26, 95% CI 0.10–0.72, respectively). NIV and NIV + HFNC also reduced ICU mortality (OR 0.40, 95% CI 0.22–0.74; OR 0.32, 95% CI 0.12–0.85). NIV + HFNC ranked best in terms of reintubation rate, respiratory failure and ICU mortality based on the surface under the cumulative ranking curve (SUCRA) (99.3, 87.1, 88.2, respectively). Although there was no significant difference in shortening ICU stay and LOS among the four methods, HFNC ranked first based on the SUCRA.

**Conclusion:**

Preventive use of NIV + HFNC after scheduled extubation is probably the most effective respiratory support method for preventing reintubation, respiratory failure and ICU death in high-risk patients with extubation failure. HFNC alone seems to be the best method to shorten ICU stay and LOS.

**Systematic review registration:**

[https://www.crd.york.ac.uk/prospero/], identifier [CRD42022340623].

## Introduction

Extubation failure still occurs in 10–20% of patients who pass a spontaneous breathing trial (SBT) and undergo planned extubation, and is associated with poor outcomes such as reintubation, prolonged duration of intensive care unit (ICU) stay and hospital stay, and increased mortality (1, 2). For patients at high-risk of extubation failure, such as those older than 65 years and those with underlying cardiopulmonary disease, the rate of reintubation can be as high as 48% (3). And the need to reintubation is related to an increased ICU mortality of 26–50% (4). In addition to the personal challenges on patients and their families, the intensive care related resources these patients receive place a significant burden on the public health system (5). Therefore, it is essential to receive prophylactic respiratory support for post-extubated patients, especially those with high risk factors.

Various respiratory management strategies have been proposed to alleviate extubation failure and reintubation. Conventional oxygen therapy (COT) is the most frequently administered respiratory support method to improve post-extubation hypoxemia. However, the delivered fraction of inspired oxygen (FiO_2_) of COT such as nasal cannulas and facemasks with reservoirs is unstable (6). And for Venturi masks, one of the COT, oxygen is passively heated and humidified (7). NIV has been recommended for patients at high-risk of reintubation, particularly those with hypercapnia (8). Nevertheless, NIV is prone to aspiration pneumonia, interface intolerance, and patient discomfort (9). High-flow nasal cannula (HFNC) is a novel device that delivers high-concentration humidified oxygen through nasal cannulas, and generates a low level of positive end-expiratory pressure (PEEP) in the upper airways, facilitating alveolar recruitment (10, 11). Moreover, HFNC improves respiratory secretions management and decreases the anatomical dead space ventilation and therefore the CO_2_ rebreathing (12). But its ability to unload respiratory muscles in high-risk patients with extubation failure may be lower than that provided by NIV (13–15).

Previous meta-analyses have shown that HFNC was superior to COT but non-inferior to NIV in reducing reintubation rates in patients with acute respiratory failure (16, 17). However, the comprehensive effectiveness of these three oxygen therapies for high-risk patients with extubation failure, such as those over 65 years old and those with underlying cardiopulmonary disease, remains unclear. In addition, the use of HFNC during NIV breaks has been introduced recently, and this sequential alternate protocols (NIV + HFNC) could prevent reintubation compared with HFNC alone (18). While the efficacy on reducing mortality in patients at high-risk of extubation failure is controversial (18, 19). Therefore, we performed this network meta-analysis (NMA) to evaluate the comprehensive efficacy of prophylactic use of various oxygen therapies (COT, NIV, HFNC, and NIV + HFNC) on reducing reintubation rate and respiratory failure after planned extubation in patients at high-risk of extubation failure.

## Methods

This NMA was reported according to the Preferred Reporting Items for Systematic Reviews and Meta-Analyses (PRISMA) extension statements for reviews incorporating network meta-analyses (Supplementary Table 1) (20). The study protocol was registered on PROSPERO (CRD42022340623).

### Search strategy

The search strategy included controlled vocabulary (i.e., Medical Subject Headings) and free-text words for three basic concepts: (1) extubation, (2) high-risk patients with extubation failure, and (3) oxygen therapy, non-invasive ventilation, and high-flow therapy. Two researchers (XZ and RW) independently searched relevant literature in PubMed, Cochrane Central Register of Controlled Trials, Web of Science, and Embase from inception to June 2022, with no language restrictions. The detailed search strategy is presented in Supplementary Table 2. In addition, reference lists of included articles were reviewed. We also tried to contact authors of conference proceedings to obtain unpublished data.

### Eligibility criteria

The inclusion criteria showed as following: (1) participants: adult patients (age ≥ 18 years) admitted to the ICU who received invasive mechanical ventilation (IMV) > 12 h, successfully passed the SBT and were ready for extubation, while were at high-risk of extubation failure (4, 13, 21); (2) interventions and comparisons: compared two of the four available devices: COT, NIV, HFNC, and NIV + HFNC. All of these methods were used for preventive purposes; (3) outcomes: the primary outcomes were reintubation rate and respiratory failure, and the second outcomes included ICU mortality, ICU stay and length of hospital stay (LOS). Studies reporting on at least one of the above outcomes were included; and (4) study design: prospective randomized controlled trials (RCTs).

The exclusion criteria included the following: (1) non-RCTs, including reviews, retrospective studies, cohort studies, and crossover studies; (2) more than half of the subjects were post-operative patients; (3) language not in English; (4) studies in which respiratory support was used for therapeutic purpose; (5) abstracts without full-text manuscripts.

According to the previous studies (4, 13, 21), “high risk” of extubation failure was defined as the presence of at least one of the following factors: (1) age > 65 years; (2) underlying cardiopulmonary disease; (3) APACHE II score > 12 at extubation; (4) body mass index > 30 kg/m^2^; (5) upper airway obstruction with stridor; (6) weak cough; (7) more than one comorbidity; (8) more than one SBT failure; (9) PaCO_2_ > 45 mmHg after extubation; and (10) duration of IMV > 7 days.

### Study selection

After filtering duplicate records, two researchers (XZ and RW) independently selected and evaluated the titles and abstracts of the retrieved literature, and then the shortlisted studies were screened again to assess their adherence to the eligibility criteria. A third reviewer (JD) participated in the discussion to adjudicate disagreements. Language was limited to English during selection.

### Data extraction

Data from eligible studies were extracted by two researchers (MM and MG) independently and combined to form a specific data collection sheet. The abstracted data included the name of the first author, publication year, number and locations of study centers, sample size, interventions and comparators, definition of high-risk patients, study outcomes, complications, main reason for intubation, and duration of mechanical ventilation before inclusion. Moreover, age, sex, acute physiology and chronic health evaluation (APACHE) II score on admission, atrial partial pressure of carbon dioxide (PaCO_2_) at the end of SBT, and oxygenation index at the end of SBT were also recorded. The disagreement was resolved by a joint review of the full text to reach consensus.

### Quality assessment

Two researchers (JD and MG) independently assessed the risk of bias for primary outcomes in eligible studies using the Cochrane Risk of Bias tool (22). Each trial was judged as low, unclear, or high risk with respect to adequate sequence generation, allocation concealment, blinding of participants and personnel, blinding of outcome assessment, incomplete outcome data, selective reporting, and other bias. We resolved disagreements by a discussion with a third reviewer (SG) to reach consensus.

### Statistical analysis

#### Direct comparison meta-analysis

A conventional pairwise meta-analysis was performed using RevMan 5.3 (RevMan 2014). Effect sizes from the forest plots were expressed as odds ratios (ORs) and mean differences (MDs), both with 95% confidence intervals (CIs), for categorical and continuous data, respectively. Outcome measures were pooled using a random effect model. A two-sided *p*-value < 0.05 was considered significant. To evaluate heterogeneity across studies within each direct comparison, we visually inspected the forest plots and quantified using the *Q* test and the *I*^2^ statistic. When heterogeneity was identified (*I*^2^ > 50%), we quantified it using the Chi-square test (*p* value). We planned to use a funnel plot for the possibility of publication bias, if ≥ 10 studies were available.

#### Geometry of the network

Network plots were constructed to determine the number of studies included in this NMA. We demonstrated the network geometry that presented the nodes as interventions and each head-to-head direct comparison as lines connecting these nodes. The size of the node was proportional to the number of trials that included in each method. The thickness of the connecting line was proportional to the number of direct comparisons.

#### Network comparison meta-analysis

A random effect NMA was performed using a frequentist framework to calculate ORs for categorical outcomes and MDs for continuous outcomes, with corresponding 95% CIs. The statistical analysis was performed using the Netmeta package in Stata/SE 16.0 (Stata-Corp, College Station, TX, USA). A two-sided *p*-value < 0.05 was considered statistically significant.

Assessment of the risk of bias across studies followed considerations on pairwise meta-analysis. The indirectness of each study included in the NMA was evaluated according to the relevance to study population, interventions, outcomes, and study setting. The approach to imprecision comprised a comparison of the range of treatment effects included in the 95% CI with the range of equivalence. We assessed the imprecision of treatment effects for a clinically important ORs of <0.8 or >1.25 in the CIs. To evaluate the heterogeneity, we compared the posterior distribution of the estimated heterogeneity variance with its predictive distribution. The concordance between assessments based on CI and prediction intervals, which do and do not capture heterogeneity, respectively, was used to assess the importance of heterogeneity. Inconsistency between direct and indirect estimates in the entire network for each outcome was assessed locally with a loop-specific approach and globally with design-by treatment interaction model (23). And publication bias was assessed visually using a funnel plot (24).

We also ranked the preventive effectiveness of each strategy according to the probability of achieving the best results through the surface under the cumulative ranking curve (SUCRA) (25). The higher the SUCRA value, which ranges from 0 to 100%, the more likely this respiratory support method is to be ranked as best.

#### Grading the quality of evidence

We evaluated the quality of evidence for each outcome using the modified Grading of Recommendation, Assessment, Development and Evaluation (GRADE) tool for NMA (26). The weight contribution matrix was constructed to assess the information contribution of direct evidence to entire NMA estimates (27). The quality of evidence in NMA would be degraded because of the risk of bias, indirectness, imprecision, publication bias, and incoherence (27).

#### Sensitive analysis

Given that small sample size and hypercapnia (PaCO_2_ > 45 mmHg) at the end of SBT might affect the relative effectiveness of respiratory support methods, two sensitivity analyses were performed to assess the robustness of NMA results to the primary outcomes by excluding studies with sample size < 50 or those involving patients with hypercapnia at the end of SBT.

## Results

### Study selection

**FIGURE 1:**
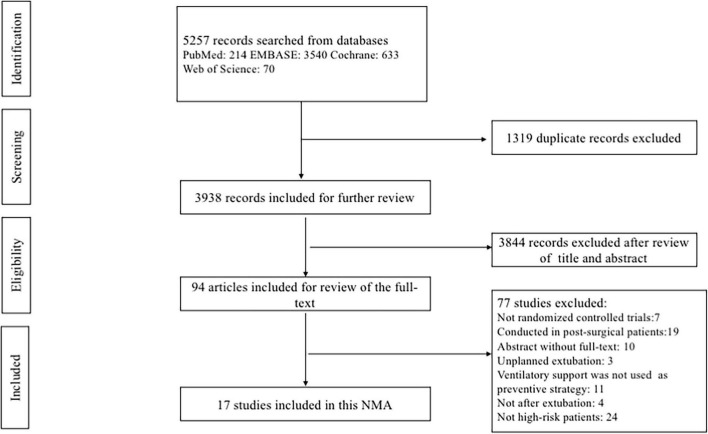
PRISMA flowchart for the study selection process. NMA, network meta-analysis.

The comprehensive database search yielded 5257 records. After excluding 1319 duplicates and 3844 irrelevant citations, we reviewed the full text of the remaining 94 records. Finally, a total of 17 eligible RCTs (3, 13, 15, 18, 19, 28–39), representing 2813 patients, were included in this NMA. A flowchart describing the detailed retrieval strategy is presented in Figure 1.

### Study characteristics

The characteristics of each study included in this NMA are summarized in Table 1 and Supplementary Table 3. All the selected studies were published between 2005 and 2022, and the sample size ranged from 29 to 641. Of the 17 included RCTs, 8 (47%) were multicenter (3, 13, 18, 19, 28–30, 33) and 9 (53%) were single-center (15, 31, 32, 34–39). Four trials (23.5%) recruited patients from Spain (3, 13, 28, 29), 4 (23.5%) from China (17, 34, 35, 38), and 3 (17.6%) from France (18, 19, 33). The definition of high-risk factors varied from study to study. Respiratory disease was the most common complication in these high-risk patients. The main reasons for intubation were chronic obstructive pulmonary disease with acute exacerbation (AECOPD) and pulmonary infection. Most patients among the trials were older than 65, with a higher mean proportion of male than female. The APACHE II score on admission was greater than 12 in 9 of 10 (90%) trials (3, 13, 28, 29, 32, 34, 37–39). The PaCO_2_ at the end of SBT was greater than 45 mmHg in 2 of 12 (16.7%) trials (3, 35). Oxygenation index at the end of SBT was mostly greater than 200 mmHg among the included studies.

**TABLE 1:** Characteristics of the studies included in the network meta-analysis.

References	Design	Contrary	Sample size	C	I	Outcome	High-risk definition	Complication	Main reason for intubation	MV before inclusion (d)
Ferrer et al. (28)	Multi-center	Spain	162	COT	NIV	1, 2, 3, 4, 5, 6	Age > 65 year, cardiac failure as the cause of intubation, or increased severity, assessed by an APACHE II score >12 on the day of extubation.	Chronic respiratory disorders (49%; 52%)	AECOPD (30.1%; 30.4%)	C:7 ± 5 I:6 ± 4
Fernandez et al. (29)	Multi-center	Spain	155	COT	HFNC	1, 2, 3, 4, 5, 6	>65 years, heart failure as cause of intubation, non-hypercapnic moderate-to-severe COPD, APACHE II score >12 points at extubation, body mass index>30 kg/m^2^, weak cough and copious secretions, more than one SBT failure, or MV>7 days.	Na	Na	C:7.4 ± 3.6 I:8.2 ± 5.9
Hernández et al., (13)	Multi-center	Spain	604	NIV	HFNC	1, 2, 3, 4, 5, 6	Age > 65 years; heart failure; moderate to severe chronic obstructive pulmonary disease; an APACHE II score > 12 on extubation day; body mass index of more than 30; airway patency problems; inability to deal with respiratory secretions; difficult or prolonged weaning; 2 or more comorbidities; and mechanical ventilation for > 7 days.	Respiratory primary failure (38.5%; 33.8%)	Na	C:4 (2–8) I:4 (2–9)
Cho et al. (15)	Single-center	Korea	60	COT	HFNC	1, 3, 4, 5, 6	Age > 65 years, APACHE II score > 12 points on extubation day, obesity, poor expectoration, airway patency problems, difficult or prolonged weaning, and more than one comorbidity.	Chronic lung disease (44.8%; 38.7%)	Pulmonary infection (80.6%; 51.7%)	C:5.7 ± 5.2 I:7.1 ± 4.7
Thille et al. (18)	Multi-center	France	641	HFNC	NIV + HFNC	1, 2, 3, 4, 5, 6	>65 years or had any underlying chronic cardiac or lung disease. Underlying chronic cardiac diseases; history of cardiogenic pulmonary edema; documented ischemic heart disease; or permanent atrial fibrillation. Underlying chronic lung diseases.	Na	Acute respiratory failure (52%; 49%)	C:5 (3–9) I:6 (3–11)
Nava et al. (30)	Multi-center	Italy	97	COT	NIV	1, 3, 4, 5, 6	More than one consecutive failure of weaning trial, Chronic heart failure, PaCO2 > 45 mm Hg after extubation, More than one comorbidity (excluding chronic heart failure), Weak cough defined as Airway Care Score values > 8 and >12, Upper airways stridor at extubation not requiring immediate reintubation	Na	AECOPD (31%; 36%)	C:7.46 ± 6 I:6.14 ± 7
Ferrer et al. (3)	Multi-center	Spain	106	COT	NIV	1, 2, 3, 4, 5, 6	At high-risk of extubation failure	COPD or chronic bronchitis (69%; 70%)	AECOPD (48%; 52%)	C:4 ± 2 I:5 ± 3
Khilnani et al. (31)	Single-center	India	40	COT	NIV	1, 6	Acute exacerbation of COPD with type-2 respiratory failure	Chronic cor pulmonale (25%; 15%)	Na	C:11 ± 4.5 I:10 ± 4.7
Ornico et al. (32)	Single-center	Brazil	38	COT	NIV	1, 4, 5	Acute respiratory failure	Na	Pneumonia (88.9%; 80%)	C:9.5 ± 6.1 I:9.9 ± 8.1
Vargas et al. (33)	Multi-center	France	143	COT	NIV	1, 2, 3, 4, 5	Patients with known or suspected chronic respiratory disorders, or those who tolerated a spontaneous breathing trial with hypercapnia defined by a PaCO_2_ > 45 mmHg.	Diabetes mellitus (33.3%; 26.7%)	AECOPD (55.5%; 56.3%)	C:6 (4–11) I:7 (5–11)
Song et al. (34)	Single-center	China	60	COT	HFNC	1	Acute respiratory failure	Na	Pneumonia (40%; 43.3%)	C:5.4 ± 2.8 I:5.5 ± 3.4
Jing et al. (35)	Single-center	China	42	NIV	HFNC	1, 2, 4, 5	AECOPD, with hypercapnia (PaCO2 >45 mmHg) at the time of extubation	Chronic cor pulmonale (90%; 86.4%)	AECOPD	C:3.4 ± 1.6 I:3.3 ± 1.6
Xu et al. (36)	Single-center	China	29	COT	NIV + HFNC	1	Patients with an LUS score ≥ 14 points	Na	Na	Na
Thille et al. (19)	Multi-center	France	410	HFNC	NIV + HFNC	1, 2, 3, 4, 5, 6	At high-risk of extubation failure	Underlying chronic cardiac disease (47%; 50%)	Acute respiratory failure (48%; 45%)	C:5 (3–10) I:7 (3–12)
Mohamed and Abdalla (37)	Single-center	Egypt	120	COT	NIV	1, 3, 5	Acute respiratory failure	COPD (31.6%; 26.6%)	Na	C:7.1 ± 1.8 I:6.2 ± 1.6
Adıyeke et al. (39)	Single-center	Turkey	50	COT	NIV	1, 2	Acute respiratory failure	Na	Na	Na
Hu et al. (38)	Single-center	China	56	COT	HFNC	2, 4, 5	Age > 65 years, congestive heart failure, COPD, bronchiectasis or old pulmonary tuberculosis with lung destruction, idiopathic pulmonary fibrosis, ESRD under maintenance dialysis, respiratory muscle weakness related to neuromuscular disease, inadequate respiratory tract secretion management ability, body mass index > 30, adult respiratory distress syndrome, or invasive MV use of > 7 days.	Hypertension (63%; 58.6%)	Respiratory tract infection (33.3%; 44.8%)	C:7 (5–11) I:9 (6–12)

C, control setting; I, intervention setting; AECOPD, chronic obstructive pulmonary disease with acute exacerbation; 1, reintubation; 2, post-extubation respiratory failure; 3, ICU mortality; 4, ICU stay; 5, length of hospital stay; COT, conventional oxygen therapy; NIV, non-invasive ventilation; HFNC, high-flow nasal catheter; MV, mechanical ventilation. The data on MV before inclusion presented as mean ± SD or median (IQR).

### Quality assessment

**FIGURE 2:**
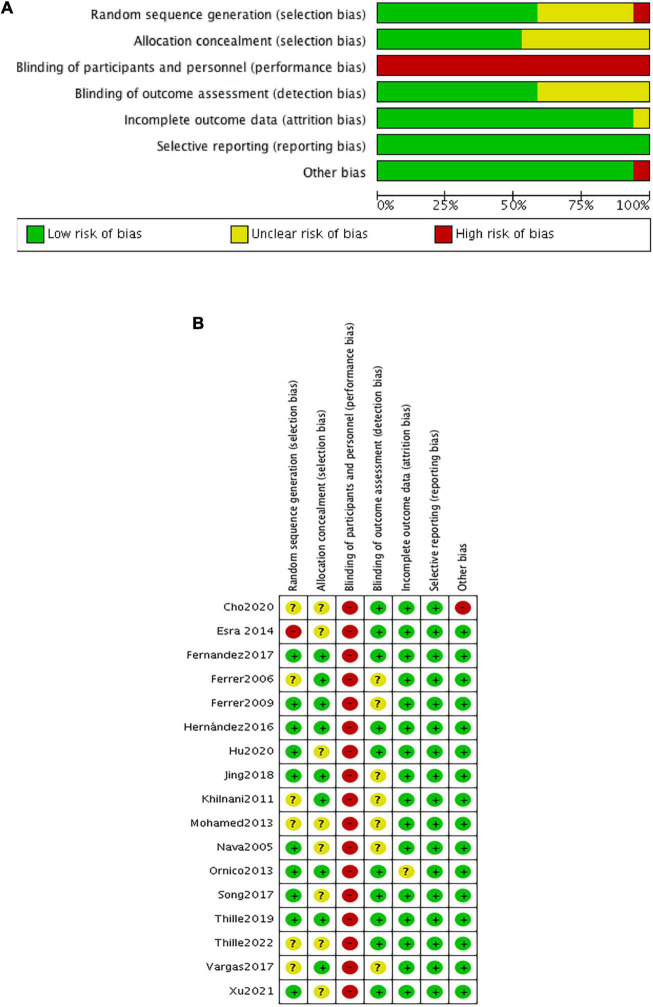
Risk of bias for each comparison. **(A)** Risk of bias summary; **(B)** risk of bias graph.

The risk of bias within eligible studies is shown in Figure 2. All trials were assessed as low or unclear risk of bias with respect to random sequence generation and allocation concealment, except for one (39) in which participants were grouped by the admission number. All studies were judged as having a high risk of performance bias because of the inability to blind caregivers to ventilation device. There were seven unclear detection bias due to the unknown definition of reintubation (3, 28, 30, 31, 33, 35, 37). Additionally, one trial (15) had a high risk of other bias associated with the imbalanced baseline.

### Pairwise meta-analysis

Compared with COT, NIV was more effective in preventing reintubation, respiratory failure, and ICU mortality. NIV + HFNC reduced the rate of reintubation and respiratory failure compared with HFNC alone. HFNC shortened the ICU stay compared with NIV (Supplementary Figures 1–5).

### Network meta-analysis

The included trials evaluated four interventions, including five head-to-head comparisons for reintubation and four head-to-head comparisons for respiratory failure (Supplementary Figure 6). There were two loops in the reintubation network plot (COT-NIV-HFNC; COT-HFNC- NIV + HFNC) (Supplementary Figure 6A). Supplementary Figure 6B showed only one loop in the network plot of respiratory failure (COT-NIV-HFNC). The weight contribution matrix and league table are shown in Supplementary Figures 7–11 and Supplementary Table 4.

### Reintubation

**FIGURE 3:**
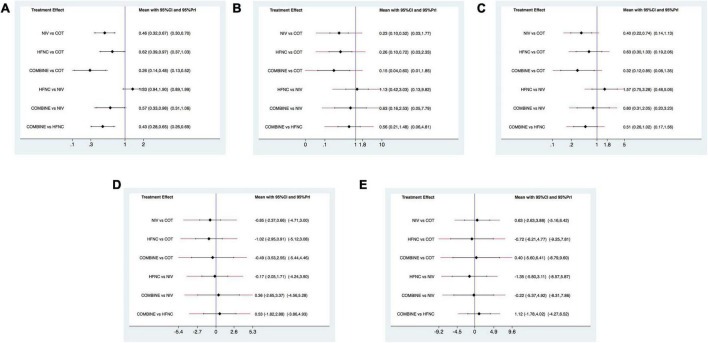
Forest plots for reintubation rate, respiratory failure, ICU mortality, ICU stay, and LOS. **(A)** Reintubation rate; **(B)** respiratory failure; **(C)** ICU mortality; **(D)** ICU stay; **(E)** LOS. ICU, intensive care unit; LOS, length of stay; NIV, non-invasive ventilation; COT, conventional oxygen therapy; HFNC, high-flow nasal cannula; CI, confidence interval; Prl, prediction interval.

**FIGURE 4:**
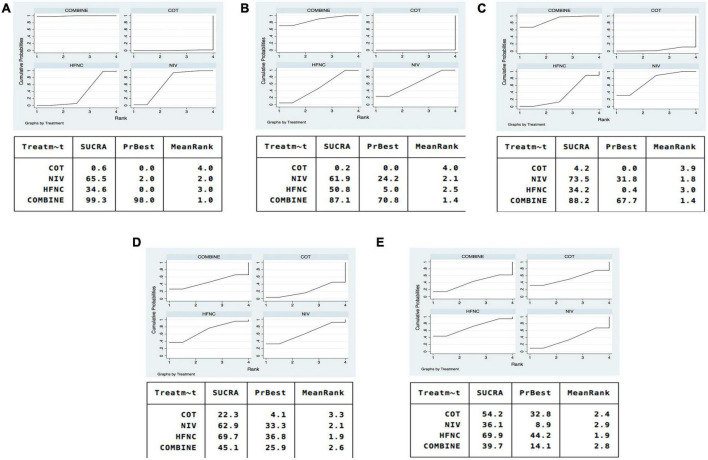
SUCRA of oxygen therapies for reintubation rate, respiratory failure, ICU mortality, ICU stay, and LOS. **(A)** Reintubation; **(B)** respiratory failure; **(C)** ICU mortality; **(D)** ICU stay; **(E)** LOS. NIV, non-invasive ventilation; COT, conventional oxygen therapy; HFNC, high-flow nasal cannula; SUCRA, surface under cumulative ranking curve.

Sixteen studies were included in the analysis of reintubation (3, 13, 15, 18, 19, 28–37, 39). All the three methods (NIV, HFNC, NIV + HFNC) were superior to COT in reintubation (OR 0.46, 95% CI 0.32–0.67; OR 0.62, 95% CI 0.39–0.97; OR 0.26, 95% CI 0.14–0.48, respectively) (Figure 3A). HFNC was comparable to NIV in reducing reintubation rate (OR 1.33, 95% CI 0.94–1.90). Compared to NIV and HFNC, NIV + HFNC prevented reintubation with significant differences (OR 0.57, 95% CI 0.33–0.98; OR 0.43, 95% CI 0.28–0.65, respectively). Figure 4A showed the treatment rankings, which revealed that the hierarchy for efficacy in reducing reintubation was NIV + HFNC (SUCRA 99.3) > NIV (SUCRA 65.5) > HFNC (SUCRA 34.6) > COT (SUCRA 0.6).

The quality of evidence for reintubation estimated by NMA was rated as low to moderate (Table 2A). The study limitation was detected for all the comparisons because of a high risk of performance bias (Figure 2). The funnel plot suggested no publication bias (Supplementary Figure 12). The imprecision of two direct comparisons (HFNC vs. COT and NIV + HFNC vs. COT) resulted in “some concern” because 95% CIs included values favoring either treatment. Quality of evidence for indirect estimates downgraded by one level for serious heterogeneity due to *I*^2^ in three comparisons (NIV vs. COT, HFNC vs. COT, and NIV + HFNC vs. COT). And heterogeneity was observed in one network comparison due to the predictive interval (HFNC vs. COT) (Figure 3A). There was no significant difference between direct and indirect comparisons (Supplementary Figures 13, 14), indicating the consistency of different studies.

**TABLE 2:** Estimate and certainly of the evidence of direct, indirect, and network comparisons.

Comparisons	No. of RCTs	Estimate of direct comparison (95% CI)	Certainly of the evidence of direct comparison	Estimate of indirect comparison (95% CI)	Certainly of the evidence of indirect comparison	Estimate of network comparison (95% CI)	Certainly of the evidence in network comparison

(A) Reintubation							
NIV vs. COT	8	0.43 (0.29, 0.65)	⊕⊕⊕○ Moderate^1^	0.62 (0.34, 1.15)	⊕⊕○○ Low ^4,5^	0.46 (0.32, 0.67)	⊕⊕⊕○ Moderate^8^
HFNC vs. COT	3	0.76 (0.34, 1.71)	⊕⊕○○ Low^1,2^	0.48 (0.32, 0.73)	⊕⊕○○ Low ^4,5^	0.62 (0.39, 0.97)	⊕⊕○○ Low ^8,9^
NIV + HFNC vs. COT	1	0.32 (0.05, 2.13)	⊕⊕○○ Low^1,2^	0.53 (0.36, 0.77)	⊕⊕○○ Low ^4,5^	0.26 (0.14, 0.48)	⊕⊕⊕○ Moderate^8^
HFNC vs. NIV	2	1.26 (0.85, 1.86)	⊕⊕⊕○ Moderate ^1^	0.46 (0.35, 0.60)	⊕⊕⊕○ Moderate^4^	1.33 (0.94, 1.90)	⊕⊕⊕○ Moderate^8^
NIV + HFNC vs. HFNC	2	0.33 (0.11, 0.97)	⊕⊕⊕○ Moderate^1^	0.59 (0.40, 0.87)	⊕⊕⊕○ Moderate^4^	0.43 (0.28, 0.65)	⊕⊕⊕○ Moderate^8^

**(B) Respiratory failure**							

NIV vs. COT	4	0.20 (0.09, 0.43)	⊕⊕⊕○ Moderate^1^	0.60 (0.32, 1.12)	⊕⊕⊕○ Moderate^4^	0.23 (0.10, 0.52)	⊕⊕○○ Low ^8,9^
HFNC vs. COT	2	0.26 (0.02, 3.60)	⊕⊕○○ Low^1,2^	0.31 (0.15, 0.62)	⊕⊕○○ Low ^4,5^	0.26 (0.10, 0.72)	⊕⊕○○ Low ^8,9^
HFNC vs. NIV	2	0.85 (0.20, 3.58)	⊕⊕○○ Low^1,2^	0.24 (0.11, 0.50)	⊕⊕○○ Low ^4,5^	1.13 (0.42, 3.03)	⊕⊕○○ Low ^8,10^
NIV + HFNC vs. HFNC	2	0.57 (0.43, 0.76)	⊕⊕⊕○ Moderate^1^	NE^6^		0.56 (0.21, 1.48)	⊕⊕○○ Low ^8,10^

**(C) ICU mortality**							

NIV vs. COT	5	0.33 (0.17, 0.62)	⊕⊕⊕○ Moderate^1^	1.09 (0.63, 1.86)	⊕⊕○○ Low ^4,5^	0.40 (0.22, 0.74)	⊕⊕○○ Low ^8,9^
HFNC vs. COT	2	0.96 (0.38, 2.44)	⊕⊕○○ Low^1,2^	0.47 (0.23, 0.93)	⊕⊕⊕○ Moderate^4^	0.63 (0.30, 1.33)	⊕⊕○○ Low ^8,10^
HFNC vs. NIV	1	1.15 (0.59, 2.24)	⊕⊕○○ Low^1,2^	0.46 (0.27, 0.79)	⊕⊕⊕○ Moderate^4^	1.57 (0.75, 3.28)	⊕⊕○○ Low ^8,10^
NIV + HFNC vs. HFNC	2	0.47 (0.18, 1.21)	⊕⊕⊕○ Moderate^1^	NE^6^		0.51 (0.26, 1.02)	⊕⊕⊕○ Moderate^8^

**(D) ICU stays**							

NIV vs. COT	6	–1.25 (–3.63, 1.13)	⊕○○○ Very low^1,2,3^	–0.83 (–1.47, –0.19)	⊕⊕⊕○ Moderate^4^	–0.85 (–2.37, 0.66)	⊕⊕○○ Low ^8,10^
HFNC vs. COT	3	0.02 (–2.00, 2.04)	⊕⊕○○ Low^1,2^	–1.16 (–2.57, 0.26)	⊕○○○ Very low^4,5,7^	–1.02 (–2.95, 0.91)	⊕⊕○○ Low ^8,10^
HFNC vs. NIV	2	–0.99 (–1.69, –0.30)	⊕⊕⊕○ Moderate^1^	–0.89 (–2.70, 0.91)	⊕○○○ Very low^4,5,7^	–0.17 (–2.05, 1.71)	⊕⊕○○ Low ^8,10^
NIV + HFNC vs. HFNC	2	0.64 (–0.48, 1.75)	⊕⊕○○ Low^1,2^	NE^6^		0.53 (–1.82, 2.88)	⊕⊕○○ Low ^8,10^

**(E) Length of in-hospital stay**							

NIV vs. COT	4	–0.66 (–3.76, 2.43)	⊕⊕○○ Low^1,2^	1.13 (–5.93, 8.20)	⊕⊕○○ Low ^4,7^	0.63 (–2.63, 3.88)	⊕⊕○○ Low ^8,10^
HFNC vs. COT	2	5.11 (–6.52, 16.73)	⊕⊕○○ Low^1,2^	–1.76 (-4.02, 0.49)	⊕⊕○○ Low ^4,7^	–0.72 (–6.21, 4.77)	⊕⊕○○ Low ^8,10^
HFNC vs. NIV	1	–3 (–6.28, 0.28)	⊕⊕○○ Low^1,2^	0.53 (–2.77, 3.83)	⊕⊕○○ Low ^4,7^	–1.35 (–5.80, 3.11)	⊕⊕○○ Low ^8,10^
NIV + HFNC vs. HFNC	2	1.19 (–1.08, 3.47)	⊕⊕○○ Low^1,2^	NE^6^		1.12 (–1.78, 4.02)	⊕⊕○○ Low ^8,10^

CI, confidence interval; COT, conventional oxygen therapy; HFNC, high-flow nasal cannula; NIV, non-invasive ventilation; NO, number; RCT, random controlled trial; ICU, intensive care unit; NE, not estimable.

^1^Quality of evidence for direct estimate rated down by one level for serious risk of bias because of the high risk of unblinding of participants and personnel in all included trials. ^2^Quality of evidence for direct estimate rated down by one level for serious imprecision because 95% CI include values favoring either treatment. ^3^Quality of evidence for direct estimate rated down by one level for serious incoherence. ^4^Quality of evidence for indirect estimate rated down by one level for serious risk of bias. ^5^Quality of evidence for indirect estimate rated down by one level for serious incoherence. ^6^Not estimable because no loop can be constructed for the two treatments in the evidence network. ^7^Quality of evidence for indirect estimate rated down by one level for serious imprecision because 95% CI include values favoring either treatment. ^8^Quality of evidence for network estimate rated down by one level for serious risk of bias. ^9^Quality of evidence for network estimate rated down by one level for serious incoherence. ^10^Quality of evidence for network estimate rated down by one level for serious imprecision because 95% CI include values favoring either treatment.

### Respiratory failure

Respiratory failure was reported in 10 trials (3, 13, 18, 19, 28, 29, 33, 35, 38, 39). The network estimates suggested that NIV, HFNC and NIV + HFNC were associated with a lower risk of respiratory failure compared with COT (OR 0.23, 95% CI 0.10–0.52; OR 0.26, 95% CI 0.10–0.72; OR 0.15, 95% CI 0.04–0.60, respectively) (Figure 3B). We found no significant difference in respiratory failure among NIV, HFNC, and NIV + HFNC (OR 1.13, 95% CI 0.42–3.03; OR 0.63, 95% CI 0.16–2.53; OR 0.56, 95% CI 0.21–1.48, respectively). Figure 4B showed that NIV + HFNC ranked first in reducing respiratory failure (SUCRA 87.1).

The quality of evidence for respiratory failure assessed by NMA was rated as low (Table 2B). There was still a high risk of performance bias in studies involving respiratory failure (Figure 2). Supplementary Figure 15 indicated no significant publication bias. Two network comparisons were heterogeneous due to the predictive interval (NIV vs. COT, and HFNC vs. COT) and the other two were imprecise due to the 95% CIs (HFNC vs. NIV, and NIV + HFNC vs. HFNC). The inconsistency test at the global and local levels showed no significant difference between direct and indirect comparisons (Supplementary Figures 16, 17).

### Intensive care unit mortality

Ten trials reported ICU mortality (3, 13, 15, 18, 19, 28–30, 33, 37). Compared with COT, NIV and NIV + HFNC reduced ICU mortality, with significant differences (OR 0.40, 95% CI 0.22–0.74; OR 0.32, 95% CI 0.12–0.85, respectively) (Figure 3C). HFNC was comparable to COT in reducing ICU mortality (OR 0.63, 95% CI 0.30–1.33). There were no significant differences in ICU mortality among NIV, HFNC, and NIV + HFNC. Figure 4C showed the treatment rankings, revealing that NIV + HFNC (SUCRA 88.2) was the best to alleviate ICU death. Radar map indicated that NIV + HFNC was the most effective method to prevent reintubation, respiratory failure, and ICU death (Supplementary Figure 18). No significant publication bias was detected (Supplementary Figure 19). The imprecision of two network comparisons (HFNC vs. COT and HFNC vs. NIV) resulted in “some concern” (Table 2C). And heterogeneity was observed in one comparison of NMA estimates (NIV vs. COT) (Figure 3C). There was no significant inconsistency in the global and local levels tests (Supplementary Figures 20, 21). The network geometry for ICU mortality is shown in Supplementary Figure 22.

### Intensive care unit stay

Thirteen trials reported the length of ICU stay (3, 13, 15, 18, 19, 28–30, 32, 33, 35, 37, 38). The network plot is shown in Supplementary Figure 23. There was no evidence for the superiority of one particular respiratory support method because all the CIs contained the null value (Figure 3D). HFNC ranked best among the four methods (SUCRA 69.7) (Figure 4D). The quality of evidence for ICU stay assessed by NMA was low (Table 2D). There was no significant difference in publication bias (Supplementary Figure 24). All the network comparisons were imprecise (Figure 3D). The inconsistency test at the global and local levels indicated no significant difference (Supplementary Figures 25, 26).

### Length of hospital stay

Length of hospital stay was reported in nine trials (3, 13, 15, 18, 19, 28–31). The network geometry is shown in Supplementary Figure 27. The network estimates provided low-quality evidence of no difference in LOS among the four methods (Figure 3E). Figure 4E suggested that HFNC was the most effective method to shorten LOS (SUCRA 69.9). No significant publication bias was detected (Supplementary Figure 28). All the network comparisons were subject to imprecision (Table 2E). There was no significant inconsistency in the test at global and local levels (Supplementary Figures 29, 30).

### Sensitivity analysis

Two sensitivity analyses were performed for the primary outcomes, exclusively including 13 trials with sample size ≥ 50 and 15 trails with PaCO_2_ ≤ 45 mmHg at the end of SBT. The results revealed that the relative effectiveness of various therapies remained similar (Supplementary Table 5), and the SUCRA rankings were comparable to those of the preliminary analysis (Supplementary Figures 31, 32).

## Discussion

In this study, NIV as well as HFNC, and NIV + HFNC significantly reduced reintubation rate and respiratory failure compared to COT. NIV and NIV + HFNC also lowered the risk of ICU death. Treatment rankings showed that NIV + HFNC scored highest in alleviating reintubation, respiratory failure, and ICU mortality. While HFNC ranked best in shortening ICU stay and LOS.

A multicenter RCT demonstrated that NIV + HFNC was effective in preventing reintubation compared with HFNC alone (18). NIV interspaced with HFNC breaks between NIV sessions is a strategy that combines the benefits of both methods: NIV for sustainable pressure support effect (32) and HFNC for increased comfort and easier clearance of secretions (13). As a result, NIV + HFNC can further improve gas exchange and decrease the work of breathing (WOB) (40). In this study, NIV + HFNC was found to be the best strategy for reducing reintubation rate, respiratory failure, and ICU mortality, which was consistent with the recommendation from the latest guidelines (41). In the ERS clinical practice guidelines, HFNC was recommended during NIV breaks in patients with acute hypoxemic respiratory failure to limit the need for prolonged NIV by maintaining adequate oxygenation and to increase patient comfort (41). However, a relevant NMA indicated that NIV + HFNC exhibited the potential to increase short-term mortality (42). The different conclusion may be related to the inclusion criteria. In the study by Zhou et al. (42), only part of the studies recruited patients at risk of extubation failure, and substantial heterogeneity was identified across the eligible trials. In addition, only one RCT (18) directly compared NIV + HFNC with HFNC in Zhou’s study, and the insufficient sample size may lead to the inconsistency between direct and indirect estimation.

In this study, NIV was superior to COT in terms of reintubation and respiratory failure. The high success rate may be attributed to the early application of NIV, immediately after programmed extubation, which kept the upper airway open and improved ventilation and oxygenation, thus preventing overload of respiratory muscles, the development of atelectasis, and respiratory distress (32). However, a recent meta-analysis concluded that NIV had no effect on reducing reintubation rate (43). In the above study (43), NIV was used as a treatment strategy for unplanned extubation patients in addition to a preventive strategy after scheduled extubation. And different from conventional pairwise meta-analyses that only include head-to-head comparisons, NMA can compare multiple treatments simultaneously by combining direct and indirect evidence and inform the relative effect of indirect comparison treatments, within a higher quality (44).

According to the latest ERS guidelines (41), NIV was recommended over HFNC after extubation for patients at high risk of extubation failure unless relative or absolute contraindications to NIV. In the current NMA, although HFNC was non-inferior to NIV in terms of reintubation and respiratory failure, NIV was beneficial to lower the risk of ICU death. It may be explained by the following: first, even though both methods can generate PEEP, the flow of HFNC only produces about 5–6 cmH_2_O PEEP throughout the respiratory cycle (45, 46). While NIV can offer different levels of PEEP according to patient’s needs. Therefore, the support effect of NIV is greater than that of HFNC. In addition, we focused on high-risk patients in the current study, such as those with underlying cardiopulmonary disease. NIV has been reported to have the greatest benefits in patients with hypercapnic respiratory failure caused by chronic obstructive pulmonary disease (COPD) (6), followed by congestive heart failure (CHF) (47). Positive pressure during inspiration reduces the WOB, and compensates for increased airway resistance. Positive pressure during expiration relieves venous return and prevents respiratory failure in patients with CHF (48). All of these effects may translate into a lower mortality among patients receiving NIV protocol. This may be another reason for the difference in mortality between the two methods.

In the current NMA, HFNC ranked first in shortening ICU stay and LOS among these oxygen treatments. This may be benefit from the fact that HFNC is more comfortable and better tolerated than oronasal mask (9). In a recent multicenter RCT by Maggiore et al., HFNC reduced the incidence of tachypnea and respiratory fatigue compared with Venturi mask, improving patient comfort (11). Although the ICU stay and LOS were comparable between the two groups in that study (11), this may be due to the use of therapeutic NIV rather than reintubation in patients with respiratory distress. In addition, patients with HFNC are not restricted by respiratory support in eating, drinking, and communication. And HFNC has a smaller contact area and well-humidified oxygen delivery, which is conducive to easy clearance of secretions and low risk of adverse effects (45). The high flow also irrigates the nasopharyngeal dead space, thus alleviating CO_2_ re-breathing. However, NMA estimates suggested that the 95% CI contained the null effect and these findings should be interpreted with caution.

Although early weaning from IMV after a successful SBT improves prognosis, reintubation is inevitable and significantly increases mortality (3). Therefore, it is important to choose an appropriate strategy to prevent reintubation, especially for high-risk patients. In clinical practice, NIV + HFNC could be used prophylactically after planned extubation to reduce the risk of reintubation and respiratory failure in high-risk patients. Once the patient’s vital signs are stable, HFNC alone should be applied as early as possible to shorten ICU stay and LOS.

The results of this study are useful for selecting an appropriate non-invasive oxygen therapy for post-extubation patients. There are still several limitations. First, the definition of high-risk patients lacks consistency. And the severity of the participants in each study is unknown, which may affect the certainty of NMA results. Second, we performed two sensitivity analyses to assess the robustness of NMA results. However, there were other effect modifiers, including the cause of intubation and duration of IMV. Unfortunately, no other sensitivity analyses were conducted given the limited information in the included studies. Third, only two RCTs directly compared HFNC to NIV, and the NMA effect size was mainly estimated by indirect evidence, which may lead to inaccurate evaluation of treatment effect. More studies are needed to provide a higher certainty of evidence. Finally, due to limited data, we didn’t consider the safety and economic benefits of each methods.

## Conclusion

In conclusion, prophylactic use of NIV + HFNC after scheduled extubation is probably the most effective respiratory support method to prevent reintubation, respiratory failure and ICU death in high-risk patients with extubation failure. Among these strategies, HFNC performed a beneficial effect on shortening ICU stay and LOS. Considering few direct comparison studies, more relevant high-quality RCTs are needed in the future.

## Data availability statement

The original contributions presented in this study are included in the article/Supplementary material, further inquiries can be directed to the corresponding author.

## Author contributions

XZ participated in designing the study, performing statistical analyses, and drafting the manuscript. RW participated in designing the study, study search, and study selection. MG contributed to data extraction and quality assessment. JD participated in quality assessment and the interpretation of data. MM contributed to data extraction. SG contributed to conception, design, manuscript revision for critical intellectual content, and supervision of the study. All authors read and approved the final manuscript.

## Abbreviations

AECOPD: chronic obstructive pulmonary disease with acute exacerbation
APACHE: acute physiology and chronic health evaluation
CIs: confidence intervals
COT: conventional oxygen therapy
FiO_2_: fraction of inspired oxygen
GRADE: grading of recommendation, assessment, development and evaluation
HFNC: high-flow nasal cannula
ICU: intensive care unit
IMV: invasive mechanical ventilation
LOS: length of stay
MDs: mean differences
NIV: non-invasive mechanical ventilation
NMA: network meta-analysis
ORs: odds ratios
PaCO_2_: atrial partial pressure of carbon dioxide
PRISMA: Preferred Reporting Items for Systematic Reviews and Meta-Analyses
RCT: randomized controlled trial
SBT: spontaneous breathing trial
SUCRA: surface under the cumulative ranking curve
WOB: work of breathing.
