# Supplementary material for: Efficacy of preventive use of oxygen therapy after planned extubation in high-risk patients with extubation failure: A network meta-analysis of randomized controlled trials
Source: Front Med (Lausanne). 2022 Oct 13;9:1026234. doi: 10.3389/fmed.2022.1026234 (PMC9608755; doi:10.3389/fmed.2022.1026234)
Supplement: Supplementary file 1 [file Data_Sheet_1.docx]

**Supplementary Information**

**Table S1 PRISMA NMA Checklist of Items to Include When Reporting A Systematic Review Involving a Network Meta-analysis**

| **Section/Topic** | **Item #** | **Checklist Item** | **Reported on Page #** |
| --- | --- | --- | --- |
| **TITLE** |  |  |  |
| Title | 1 | Identify the report as a systematic review *incorporating a network meta-analysis (or related form of meta-analysis).* | 1 |
|  |  |  |  |
| **ABSTRACT** |  |  |  |
| Structured summary | 2 | Provide a structured summary including, as applicable:  **Background:** main objectives  **Methods:** data sources; study eligibility criteria, participants, and interventions; study appraisal; and *synthesis methods, such as network meta-analysis.*  **Results:** number of studies and participants identified; summary estimates with corresponding confidence/credible intervals; *treatment rankings may also be discussed. Authors may choose to summarize pairwise comparisons against a chosen treatment included in their analyses for brevity.*  **Discussion/Conclusions:** limitations; conclusions and implications of findings.  **Other:** primary source of funding; systematic review registration number with registry name. | 2-3 |
|  |  |  |  |
| **INTRODUCTION** |  |  |  |
| Rationale | 3 | Describe the rationale for the review in the context of what is already known*, including mention of why a network meta-analysis has been conducted.* | 4 |
| Objectives | 4 | Provide an explicit statement of questions being addressed, with reference to participants, interventions, comparisons, outcomes, and study design (PICOS). | 5 |
|  |  |  |  |
| **METHODS** |  |  |  |
| Protocol and registration | 5 | Indicate whether a review protocol exists and if and where it can be accessed (e.g., Web address); and, if available, provide registration information, including registration number. | 5-6 |
| Eligibility criteria | 6 | Specify study characteristics (e.g., PICOS, length of follow-up) and report characteristics (e.g., years considered, language, publication status) used as criteria for eligibility, giving rationale. *Clearly describe eligible treatments included in the treatment network, and note whether any have been clustered or merged into the same node (with justification).* | 6-7 |
| Information sources | 7 | Describe all information sources (e.g., databases with dates of coverage, contact with study authors to identify additional studies) in the search and date last searched. | 6 |
| Search | 8 | Present full electronic search strategy for at least one database, including any limits used, such that it could be repeated. | 6 and table S2 |
| Study selection | 9 | State the process for selecting studies (i.e., screening, eligibility, included in systematic review, and, if applicable, included in the meta-analysis). | 7 |
| Data collection process | 10 | Describe method of data extraction from reports (e.g., piloted forms, independently, in duplicate) and any processes for obtaining and confirming data from investigators. | 7 |
| Data items | 11 | List and define all variables for which data were sought (e.g., PICOS, funding sources) and any assumptions and simplifications made. | 8 |
| **Geometry of the network** | **S1** | Describe methods used to explore the geometry of the treatment network under study and potential biases related to it. This should include how the evidence base has been graphically summarized for presentation, and what characteristics were compiled and used to describe the evidence base to readers. | 9 |
| Risk of bias within individual studies | 12 | Describe methods used for assessing risk of bias of individual studies (including specification of whether this was done at the study or outcome level), and how this information is to be used in any data synthesis. | 8 |
| Summary measures | 13 | State the principal summary measures (e.g., risk ratio, difference in means). *Also describe the use of additional summary measures assessed, such as treatment rankings and surface under the cumulative ranking curve (SUCRA) values, as well as modified approaches used to present summary findings from meta-analyses.* | 9-10 |
| Planned methods of analysis | 14 | Describe the methods of handling data and combining results of studies for each network meta-analysis. This should include, but not be limited to:   - *Handling of multi-arm trials;* - *Selection of variance structure;* - *Selection of prior distributions in Bayesian analyses; and* - *Assessment of model fit.* | 9-10 |
| **Assessment of Inconsistency** | **S2** | Describe the statistical methods used to evaluate the agreement of direct and indirect evidence in the treatment network(s) studied. Describe efforts taken to address its presence when found. | 10 |
| Risk of bias across studies | 15 | Specify any assessment of risk of bias that may affect the cumulative evidence (e.g., publication bias, selective reporting within studies). | 9 |
| Additional analyses | 16 | Describe methods of additional analyses if done, indicating which were pre-specified. This may include, but not be limited to, the following:   - Sensitivity or subgroup analyses; - Meta-regression analyses; - *Alternative formulations of the treatment network; and* - *Use of alternative prior distributions for Bayesian analyses (if applicable).* | 10-11 |
| **RESULTS†** |  |  |  |
| Study selection | 17 | Give numbers of studies screened, assessed for eligibility, and included in the review, with reasons for exclusions at each stage, ideally with a flow diagram. | 11 |
| **Presentation of network structure** | **S3** | Provide a network graph of the included studies to enable visualization of the geometry of the treatment network. | Fig. S6 |
| **Summary of network geometry** | **S4** | Provide a brief overview of characteristics of the treatment network. This may include commentary on the abundance of trials and randomized patients for the different interventions and pairwise comparisons in the network, gaps of evidence in the treatment network, and potential biases reflected by the network structure. | 12-13 |
| Study characteristics | 18 | For each study, present characteristics for which data were extracted (e.g., study size, PICOS, follow-up period) and provide the citations. | 11-12 and Table 1, S3 |
| Risk of bias within studies | 19 | Present data on risk of bias of each study and, if available, any outcome level assessment. | 12 |
| Results of individual studies | 20 | For all outcomes considered (benefits or harms), present, for each study: 1) simple summary data for each intervention group, and 2) effect estimates and confidence intervals. *Modified approaches may be needed to deal with information from larger networks.* | 13-16 |
| Synthesis of results | 21 | Present results of each meta-analysis done, including confidence/credible intervals. *In larger networks, authors may focus on comparisons versus a particular comparator (e.g. placebo or standard care), with full findings presented in an appendix. League tables and forest plots may be considered to summarize pairwise comparisons.* If additional summary measures were explored (such as treatment rankings), these should also be presented. | Fig. S1-5  Table S4  13-16 |
| **Exploration for inconsistency** | **S5** | Describe results from investigations of inconsistency. This may include such information as measures of model fit to compare consistency and inconsistency models, *P* values from statistical tests, or summary of inconsistency estimates from different parts of the treatment network. | 13-16  Fig. S13,17,21,26,30 |
| Risk of bias across studies | 22 | Present results of any assessment of risk of bias across studies for the evidence base being studied. | 13-16  Table 2 |
| Results of additional analyses | 23 | Give results of additional analyses, if done (e.g., sensitivity or subgroup analyses, meta-regression analyses*, alternative network geometries studied, alternative choice of prior distributions for Bayesian analyses,* and so forth). | 16 |
|  |  |  |  |
| **DISCUSSION** |  |  |  |
| Summary of evidence | 24 | Summarize the main findings, including the strength of evidence for each main outcome; consider their relevance to key groups (e.g., healthcare providers, users, and policy-makers). | 17 |
| Limitations | 25 | Discuss limitations at study and outcome level (e.g., risk of bias), and at review level (e.g., incomplete retrieval of identified research, reporting bias). *Comment on the validity of the assumptions, such as transitivity and consistency. Comment on any concerns regarding network geometry (e.g., avoidance of certain comparisons).* | 19-20 |
| Conclusions | 26 | Provide a general interpretation of the results in the context of other evidence, and implications for future research. | 20 |
|  |  |  |  |
| **FUNDING** |  |  |  |
| Funding | 27 | Describe sources of funding for the systematic review and other support (e.g., supply of data); role of funders for the systematic review. This should also include information regarding whether funding has been received from manufacturers of treatments in the network and/or whether some of the authors are content experts with professional conflicts of interest that could affect use of treatments in the network. | 24 |

**Table S2 Search strategy**

| **Database** | **Research equation** | **Results** |
| --- | --- | --- |
| **PubMed** | 1# ((((Cannula[MeSH Terms]) OR (Nasal Cannula)) OR (Cannula, Nasal)) OR (Nasal Cannulae)) OR (Cannulae, Nasal) (15262 records)  2# (high-flow) OR high flow (168738 records)  3# (#1 AND #2) (2418 records)  4# ((HFNC) OR (HHFNC)) OR (HHFN) (955 records)  5# ((((((oxygen inhalation therapy[MeSH Terms]) OR (Inhalation Therapy, Oxygen)) OR (Inhalation Therapies, Oxygen)) OR (Oxygen Inhalation Therapies)) OR (Therapies, Oxygen Inhalation)) OR (Therapy, Oxygen Inhalation)) OR (oxygen therapy) (110242 records)  6# #2 AND #5 (4055 records)  7# #3 OR #4 OR #6 (5195 records)  8# ((((((((((non-invasive ventilation[MeSH Terms]) OR (Noninvasive Ventilation*)) OR (Ventilation*, Noninvasive)) OR (Non-Invasive Ventilation*)) OR (Ventilation*, Non-Invasive)) OR (Ventilation*, Non-Invasive)) OR (Non Invasive Ventilation*)) OR (Ventilation*, Non Invasive)) OR (noninvasive positive pressure ventilation)) OR (non-invasive positive pressure ventilation)) OR (NIPPV) (13896 records)  9# (((conventional oxygen therapy) OR COT) OR standard oxygen therapy) OR SOT (17172 records)  10# ((venturi mask) OR face mask) OR bag valve mask (7538 records)  11# #5 OR #9 OR #10 (122770 records)  12# #7 OR #8 OR #11 (134427 records)  13# ((((((((((((((Airway Extubation[MeSH Terms]) OR Airway Extubation*) OR Extubation*, Airway) OR Tracheal Extubation*) OR Extubation*, Tracheal) OR Extubation*, Intratracheal) OR Intratracheal Extubation*) OR Endotracheal Extubation*) OR Extubation*, Endotracheal) OR postextubation) OR post-extubation) OR after extubation*) OR following extubation*) OR extubated) OR extubation* (14172 records)  14# ((((((Critical Care[MeSH Terms]) OR intensive care[MeSH Terms]) OR Critical Illness[MeSH Terms]) OR Intensive Care Units[MeSH Terms])) OR ((((((((Critical Care) OR intensive care) OR Critical* illness) OR Intensive Care Unit*) OR ICU) OR ICUs) OR intensive illness) OR critically ill)) (568213 records)  15# #12 AND #13 AND #14 Filters: Clinical Trial; Humans (214 records) | 214 |
| **Cochrane Central Register of Controlled Trials** | 1# MeSH descriptor: [Cannula] explode all trees (177 records)  2# (Nasal Cannula) OR (Cannula*, Nasal) OR (Nasal Cannula*) (2027 records)  3# #1 OR #2 (2051 records)  4# (high-flow) OR (high flow) (13338 records)  5# #3 AND #4 (1231 records)  6# (HFNC) OR (HHFNC) OR (HHFN) (616 records)  7# MeSH descriptor: [Oxygen Inhalation Therapy] explode all trees (1736 records)  8# (Inhalation Therapy, Oxygen) OR (Inhalation Therapies, Oxygen) OR (Oxygen Inhalation Therapies) OR (Therapies, Oxygen Inhalation) OR ((Therapy, Oxygen Inhalation) OR (oxygen therapy)) (22644 records)  9# #7 OR #8 (22721 records)  10# #4 AND #9 (1770 records)  11# (Noninvasive Ventilation*) OR (Ventilation*, Noninvasive) OR (Non-Invasive Ventilation*) OR (Ventilation*, Non-Invasive) OR (Ventilation*, Non-Invasive) OR (Non Invasive Ventilation*) OR (Ventilation*, Non Invasive) OR (noninvasive positive pressure ventilation) OR (non-invasive positive pressure ventilation) OR (NIPPV) (4048 records)  12# MeSH descriptor: [Noninvasive Ventilation] explode all trees (334 records)  13# #11 OR #12 (4119 records)  14# (conventional oxygen therapy) OR (COT) OR (standard oxygen therapy) OR (SOT) OR (venturi mask) OR (face mask) OR (bag valve mask) (8449 records)  15# #9 OR #14 (25767 records)  16# #5 OR #6 OR #10 OR #13 OR #15 (28766 records)  17# MeSH descriptor: [Airway Extubation] explode all trees (281 records)  18# (Airway Extubation*) OR (Extubation*, Airway) OR (Tracheal Extubation*) OR (Extubation*, Tracheal) OR (Extubation*, Intratracheal) OR (Intratracheal Extubation*) OR (Endotracheal Extubation*) OR (Extubation*, Endotracheal) OR (postextubation) OR (post-extubation) OR (after extubation*) OR (following extubation*) OR (extubated) OR (extubation*) (8121 records)  19# #17 OR #18 (8121 records)  20# MeSH descriptor: [Critical Illness] explode all trees (2606 records)  21# MeSH descriptor: [Intensive Care Units] explode all trees (4081 records)  22# MeSH descriptor: [Critical Care] explode all trees (2217 records)  23# (Critical Care) OR (intensive care) OR (Critical Illness) OR (Critical* illness) OR (Intensive Care Unit*) OR (ICU) OR (ICUs) OR (intensive illness) OR (critically ill) (61879 records)  24# #20 OR #21 OR #22 OR #23 (62112 records)  25# #16 AND #19 AND #24 Limits: in Trials (633 records) | 633 |
| **Web of Science** | 1# TS= (Nasal Cannula) OR TS= (Cannula, Nasal) OR TS= (Nasal Cannulae) OR TS= (Cannulae, Nasal) (3871 records)  2# TS=(high-flow) OR TS=(high flow) (803446 records)  3# #1 AND #2 (2652 records)  4# TS=(HFNC) OR TS=(HFNC) OR TS=(HHFN) (915 records)  5# TS=(oxygen inhalation therapy) OR TS=(Inhalation Therapy, Oxygen) OR TS=(Inhalation Therapies, Oxygen) OR TS=(Oxygen Inhalation Therapies) OR TS=(Therapies, Oxygen Inhalation) OR TS=(Therapy, Oxygen Inhalation) OR TS=(oxygen therapy) (69096 records)  6# #2 AND #5 (3894 records)  7# #3 OR #4 OR #6 (5247 records)  8# TS=(conventional oxygen therapy) OR TS=(COT) OR TS=(standard oxygen therapy) OR TS=(SOT) OR TS=(venturi mask) OR TS=(face mask) OR TS=(bag valve mask) OR TS=(oxygen inhalation therapy) OR TS=(Inhalation Therapy, Oxygen) OR TS=(Inhalation Therapies, Oxygen) OR TS=(Oxygen Inhalation Therapies) OR TS=(Therapies, Oxygen Inhalation) OR TS=(Therapy, Oxygen Inhalation) OR TS=(oxygen therapy) OR TS=(non-invasive ventilation) OR TS=(Noninvasive Ventilation*) OR TS=(Ventilation*, Noninvasive) OR TS=(Non-Invasive Ventilation*) OR TS=(Ventilation*, Non-Invasive) OR TS=(Ventilation*, Non-Invasive) OR TS=(Non Invasive Ventilation*) OR TS=(Ventilation*, Non Invasive) OR TS=(noninvasive positive pressure ventilation) OR TS=(non-invasive positive pressure ventilation) OR TS=(NIPPV) (113451 records)  9# #7 OR #8 (114356 records)  10# TS=(Airway Extubation) OR TS=(Airway Extubation*) OR TS=(Extubation*, Airway) OR TS=(Tracheal Extubation*) OR TS=(Extubation*, Tracheal) OR TS=(Extubation*, Intratracheal) OR TS=(Intratracheal Extubation*) OR TS=(Endotracheal Extubation*) OR TS=(Extubation*, Endotracheal) OR TS=(postextubation) OR TS=(post-extubation) OR TS=(after extubation*) OR TS=(following extubation*) OR TS=(extubated) OR TS=(extubation*) (14592 records)  11# TS=(Critical Care) OR TS=(intensive care) OR TS=(Critical Illness) OR TS=(Intensive Care Unit*) OR TS=(Critical* illness) OR TS=(ICU) OR TS=(ICUs) OR TS=(intensive illness) OR TS=(critically ill) (370610 records)  12# #9 AND #10 AND #11 (870 records) | 870 |
| **Embase** | 1# ('cannula'/exp OR 'cannula' OR 'nasal cannula'/exp OR 'nasal cannula' OR 'cannula, nasal' OR 'nasal cannulae' OR 'cannulae, nasal') AND ('high flow') (5934 records)  2# ('oxygen inhalation therapy'/exp OR 'oxygen inhalation therapy' OR 'inhalation therapy, oxygen' OR 'inhalation therapies, oxygen' OR 'oxygen inhalation therapies' OR 'therapies, oxygen inhalation' OR 'therapy, oxygen inhalation' OR 'oxygen therapy'/exp OR 'oxygen therapy') AND ('high flow') (3295 records)  3# 'hfnc' OR 'hhfnc' OR 'hhfn' (1862 records)  4# #1 OR #2 OR #3 (6981 records)  5# 'conventional oxygen therapy' OR 'cot' OR 'standard oxygen therapy' OR 'sot' OR 'venturi mask'/exp OR 'venturi mask' OR 'face mask'/exp OR 'face mask' OR 'bag valve mask'/exp OR 'bag valve mask' OR 'oxygen inhalation therapy':au OR 'inhalation therapy, oxygen':au OR 'inhalation therapies, oxygen':au OR 'oxygen inhalation therapies':au OR 'therapies, oxygen inhalation':au OR 'therapy, oxygen inhalation':au OR 'oxygen therapy' (88933 records)  6# 'non-invasive ventilation'/exp OR 'non-invasive ventilation' OR 'noninvasive ventilation*' OR 'ventilation*, noninvasive' OR 'non-invasive ventilation*' OR 'ventilation*, non-invasive' OR 'non invasive ventilation*' OR 'ventilation*, non invasive' OR 'noninvasive positive pressure ventilation'/exp OR 'noninvasive positive pressure ventilation' OR 'non-invasive positive pressure ventilation' OR 'nippv' (26532 records)  7# #4 OR #5 OR #6 (110972 records)  8# 'airway extubation'/exp OR 'airway extubation' OR 'airway extubation*' OR 'extubation*, airway' OR 'tracheal extubation*' OR 'extubation*, tracheal' OR 'extubation*, intratracheal' OR 'intratracheal extubation*' OR 'endotracheal extubation*' OR 'extubation*, endotracheal' OR 'postextubation' OR 'post-extubation' OR 'after extubation*' OR 'following extubation*' OR 'extubated' OR 'extubation*' (37246 records)  9# 'critical care'/exp OR 'critical care' OR 'intensive care'/exp OR 'intensive care' OR 'critical illness'/exp OR 'critical illness' OR 'critical* illness' OR 'intensive care unit*' OR 'icu' OR 'icus' OR 'intensive illness' OR 'critically ill'/exp OR 'critically ill' (1453645 records)  10# #7 AND #8 AND #9 AND 'human'/de (3540 records) | 3540 |

**Table S3 Detailed characteristics of each included study**

| Reference | Age (years) | | Female (n, %) | | APACHE II/SAPS II score on admission | | PaCO_2_ at the end of SBT (mmHg) | | Oxygenation index at the end of SBT (mmHg) | |
| --- | --- | --- | --- | --- | --- | --- | --- | --- | --- | --- |
|  | Control | Intervention | Control (n, %) | Intervention | Control | Intervention | Control | Intervention | Control | Intervention |
| Ferrer  2006 | 70±11 | 72±10 | 59 (71) | 56 (71) | 20±6^a^ | 22±5 ^a^ | 42±9 | 44±10 | 231±65 | 227±70 |
| Fernandez  2017 | 69.7±13.0 | 67.3±12.1 | 22 (29) | 32 (41) | 21±8.2 ^a^ | 21±8.8 ^a^ | Na | Na | Na | Na |
| Hernández2016 | 64.4±15.8 | 64.6±15.4 | 112 (36) | 104 (36) | 16 (14-21) ^a^ | 16(14-22) ^a^ | 39±3.2 | 41±2.2 | 194±37 | 191±34 |
| Cho  2020 | 76.9±6.5 | 78.8±7.8 | 7 (24) | 15 (48) | Na | Na | 35.2±5.4 | 38.5±7.5 | 297±119 | 272±99 |
| Thille  2019 | 70±10 | 69±10 | 107 (35) | 109 (32) | 55±17^b^ | 55±20 ^b^ | 39±8 | 40±9 | 274±93 | 275±89 |
| Nava  2005 | 53.2±19.5 | 56.0±19.3 | 19 (39) | 17 (35) | 32.5±2.6 ^b^ | 31.4±0.3 ^b^ | 39.4±5.7 | 41.7±4.3 | 270±73 | 247±60 |
| Ferrer  2009 | 70±9 | 67±10 | 16 (31) | 10 (24) | 20±7 ^a^ | 20±7 ^a^ | 53±5 | 55±6 | 206±40 | 198±39 |
| Khilnani  2011 | 58.4±7.4 | 62.1±9.0 | Na | Na | Na | Na | Na | Na | Na | Na |
| Ornico  2013 | 49±22 | 51±18 | 6 (33) | 6 (30) | 15±6 ^a^ | 17±7 ^a^ | Na | Na | Na | Na |
| Vargas  2017 | 65±13 | 64±13 | 23 (32) | 29 (41) | 45 (35–62) ^b^ | 46 (38–59) ^b^ | 41 (36-47) | 43 (36-48) | 197 (174–216) | 198 (172–244 |
| Song  2017 | 71±13 | 66±14 | 12 (40) | 14 (47) | 12.3±3.3 ^a^ | 12.9±3.0 ^a^ | Na | Na | Na | Na |
| Jing  2018 | 73.9±6.9 | 77.4±6.8 | Na | Na | 10.4±2.5 ^a^ | 11.8±3.1 ^a^ | 53.7±8.6 | 53.2±6.7 | 251±76 | 239±81 |
| Xu  2021 | Na | Na | Na | Na | Na | Na | 43.7±4.8 | 44.2±6.1 | 214±28 | 226±33 |
| Thille  2022 | 71±9 | 69±11 | 70 (34) | 71 (35) | 56 ± 18 ^b^ | 56 ± 20 ^b^ | 40±8 | 40±8 | 273±97 | 268±83 |
| Mohamed  2013 | 68.9±7.2 | 64.1±6.6 | 26 (43) | 21 (35) | 21.8± 6.1 ^a^ | 23.2±5.4 ^a^ | 42.8 ±7.7 | 39.9±6.3 | Na | Na |
| Hu  2020 | 74.9±11.4 | 72.9±13.1 | 10 (37) | 7 (24) | 25 (22–30) ^a^ | 27 (23–29) ^a^ | 38±7.4 | 42±7.9 | 279±91 | 320±90 |
| Esra  2014 | 67.8±17.3 | 71.9±11.0 | 15 (60) | 12 (48) | 20.8±7.0 ^a^ | 19.5±4.5 | Na | Na | Na | Na |

The data on age, APACHE II/SAPS II score, PaCO2, and PaO2:FiO2 ratio are presented as mean ± SD or median (IQR)

a APACHE II score on admission

b SAPS II score on admission

**Table S4 League table for networks of respiratory support methods**

| Re-intubation rate (OR with 95% CI) | | | |
| --- | --- | --- | --- |
| **COT** | 0.46 (0.32,0.67) | 0.62 (0.39,0.97) | 0.26 (0.14,0.48) |
|  | **NIV** | 1.33 (0.94,1.90) | 0.57 (0.33,0.98) |
|  |  | **HFNC** | 0.43 (0.28,0.65) |
|  |  |  | **NIV+HFNC** |
| Respiratory failure (OR with 95% CI) | | | |
| **COT** | 0.23 (0.10,0.52) | 0.26 (0.10,0.72) | 0.15 (0.04,0.60) |
|  | **NIV** | 1.13 (0.42,3.03) | 0.63 (0.16,2.53) |
|  |  | **HFNC** | 0.56 (0.21,1.48) |
|  |  |  | **NIV+HFNC** |
| ICU mortality (OR with 95% CI) | | | |
| **COT** | 0.40 (0.22,0.74) | 0.63 (0.30,1.33) | 0.32 (0.12,0.85) |
|  | **NIV** | 0.64 (0.30,1.34) | 0.80 (0.31,2.05) |
|  |  | **HFNC** | 0.51 (0.26,1.02) |
|  |  |  | **NIV+HFNC** |
| ICU stay (MD with 95%CI) | | | |
| **COT** | -0.85 (-2.37, 0.66) | -1.02 (-2.95, 0.91) | -0.49 (-3.53,2.55) |
|  | **NIV** | -0.17 (-2.05, 1.71) | 0.36 (-2.65,3.37) |
|  |  | **HFNC** | 0.53(-1.82, 2.88) |
|  |  |  | **NIV+HFNC** |
| Length of hospital stay (MD with 95%CI) | | | |
| **COT** | 0.63 (-2.63,3.88) | -0.72 (-6.21,4.77) | 0.40 (-5.60,6.41) |
|  | **NIV** | -1.35 (-5.80,3.11) | -0.22 (-5.37,4.92) |
|  |  | **HFNC** | 1.12 (-1.78,4.02) |
|  |  |  | **NIV+HFNC** |

The column treatment is compared with the row treatment.

COT conventional oxygen therapy, NIV noninvasive ventilation, HFNC high flow nasal catheter, ICU intensive care unit, OR odds ratio, MD mean difference, CI confidence interval

**Table S5 Sensitivity analysis for the primary outcomes**

| Comparisons | After excluding trials with sample size < 50 | | | | After excluding trials with PaCO_2_ > 45 mmHg at the end of SBT | | | |
| --- | --- | --- | --- | --- | --- | --- | --- | --- |
|  | No. of RCTs | Estimate of direct comparison  (95% CI) | Estimate of indirect comparison  (95% CI) | Estimate of network comparison  (95% CI) | No. of RCTs | Estimate of direct comparison  (95% CI) | Estimate of indirect comparison  (95% CI) | Estimate of network comparison  (95% CI) |
| (a) Reintubation | | | | | | | | |
| NIV vs. COT | 6 | 0.45  (0.30, 0.70) | 1.12  (0.76, 1.65) | 0.48  (0.33, 0.71) | 7 | 0.43  (0.27, 0.68) | 0.60  (0.32, 1.14) | 0.46  (0.30, 0.70) |
| HFNC vs. COT | 3 | 0.76  (0.34, 1.71) | 0.58  (0.36, 0.96) | 0.63  (0.39, 1.01) | 3 | 0.76  (0.34, 1.71) | 0.47  (0.29, 0.5) | 0.62  (0.37, 1.05) |
| HFNC vs. NIV | 1 | 1.25  (0.84, 1.85) | 0.51  (0.35, 0.75) | 1.31  (0.91, 1.87) | 1 | 1.25  (0.84, 1.85) | 0.47  (0.35, 0.63) | 1.35  (0.84, 2.17) |
| NIV+HFNC vs. HFNC | 2 | 0.33  (0.11, 0.97) | NE | 0.42  (0.27, 0.65) | 2 | 0.34  (0.10, 1.14) | 0.58  (0.38, 0.90) | 0.44  (0.24, 0.79) |
| NIV+HFNC vs. COT | NE | NE | NE | NE | 1 | 0.26 (0.14,0.48) | 0.52  (0.34, 0.80) | 0.27  (0.13, 0.55) |
| (b) Respiratory failure | | | | | | | | |
| NIV vs. COT | 4 | 0.20  (0.09, 0.43) | 0.55  (0.32, 0.95) | 0.24 (0.11,0.55) | 3 | 0.20  (0.06, 0.67) | 0.59  (0.45, 0.77) | 0.25 (0.09,0.75) |
| HFNC vs. COT | 2 | 0.26  (0.02, 3.60) | 0.26  (0.13, 0.53) | 0.24 (0.08,0.70) | 2 | 0.26  (0.02, 3.60) | 0.44  (0.28, 0.68) | 0.24 (0.06,0.86) |
| HFNC vs. NIV | 1 | 0.56  (0.39, 0.78) | 0.24  (0.11, 0.50) | 0.98 (0.34,2.83) | 1 | 0.56  (0.39, 0.78) | 0.39  (0.23, 0.68) | 0.93 (0.26,3.35) |
| NIV+HFNC vs. HFNC | 2 | 0.57  (0.43, 0.76) | NE | 0.56 (0.21,1.52) | 2 | 0.62  (0.40, 0.94) | NE | 0.61 (0.18,2.04) |

CI confidence interval, COT conventional oxygen therapy, HFNC high-flow nasal cannula, NIV noninvasive ventilation, NO. number, RCT, random controlled trial, NE not estimable, SBT spontaneous breathing trial, PaCO_2_ atrial partial pressure of carbon dioxide.

(a) NIV vs COT


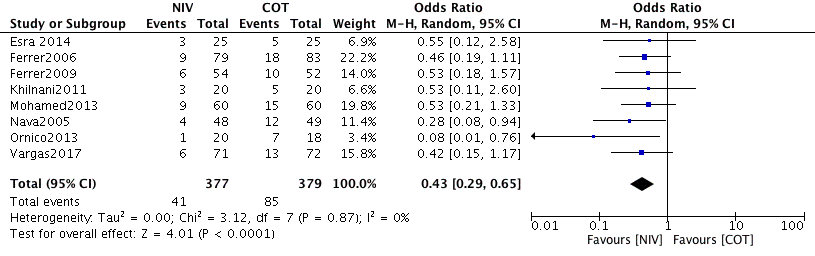


(b) HFNC vs COT


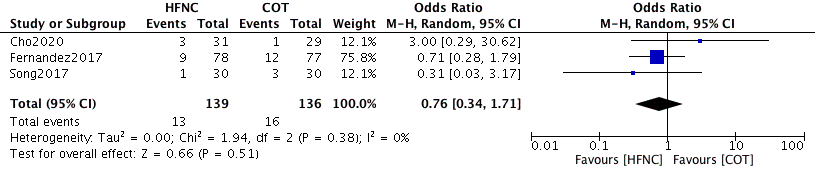


(c) HFNC vs NIV


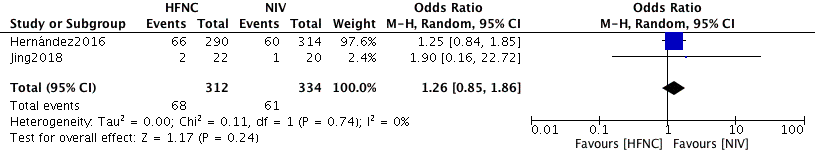


(d) NIV+HFNC vs COT


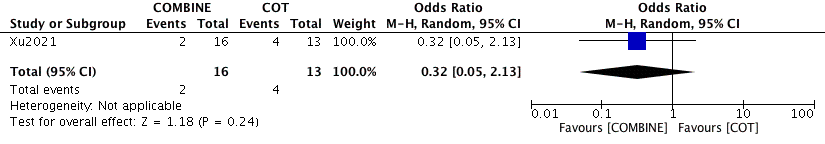


(e) NIV+HFNC vs HFNC


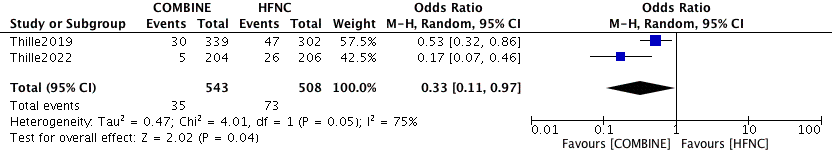


**Figure S1. Forest plots for the pairwise comparison of reintubation rate.**

COT conventional oxygen therapy, NIV noninvasive ventilation, HFNC high flow nasal catheter

(a) NIV vs COT


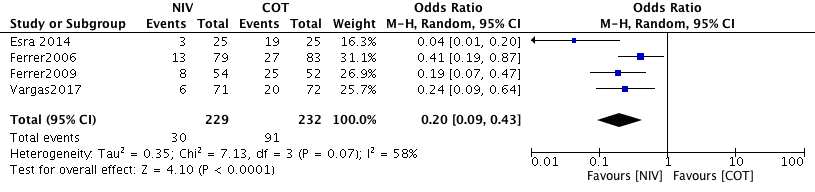


(b) HFNC vs COT


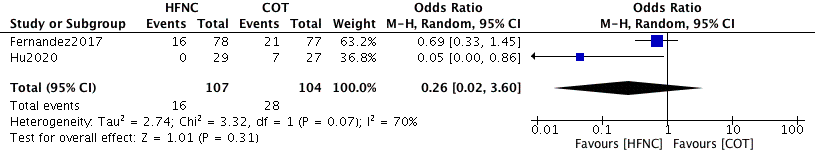


(c) HFNC vs NIV


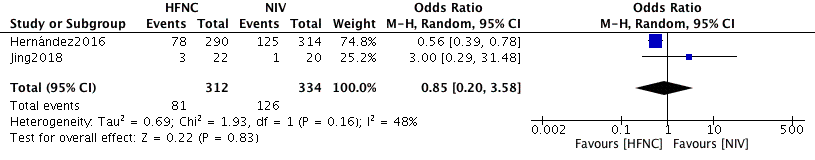


(d) NIV+HFNC vs HFNC


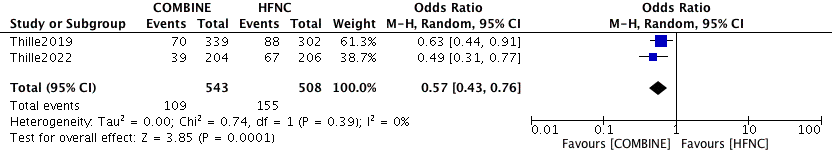


**Figure S2. Forest plots for the pairwise comparison of post-extubation respiratory failure.**

COT conventional oxygen therapy, NIV noninvasive ventilation, HFNC high flow nasal catheter

(a) NIV vs COT


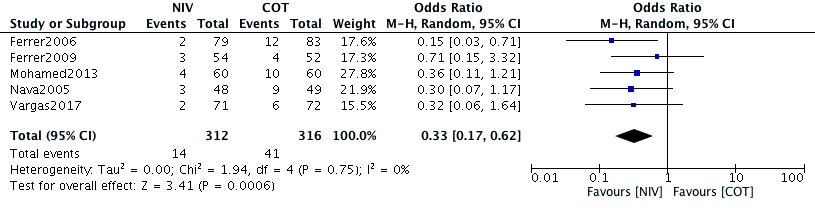


(b) HFNC vs COT


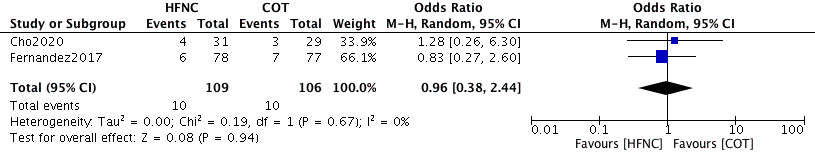


(c) HFNC vs NIV


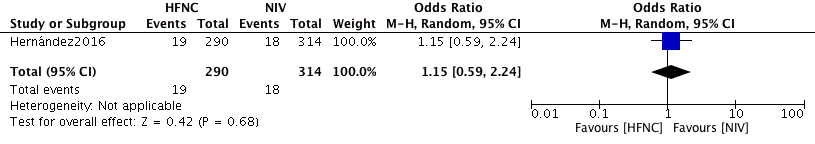


(d) NIV+HFNC vs HFNC


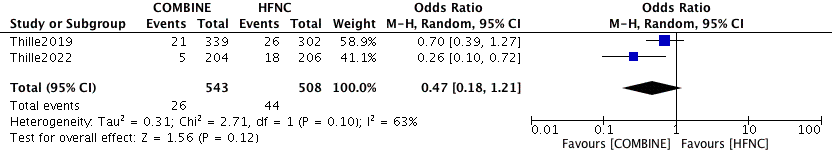


**Figure S3. Forest plots for the pairwise comparison of ICU mortality.**

COT conventional oxygen therapy, NIV noninvasive ventilation, HFNC high flow nasal catheter, ICU intensive care unit

(a) NIV vs COT


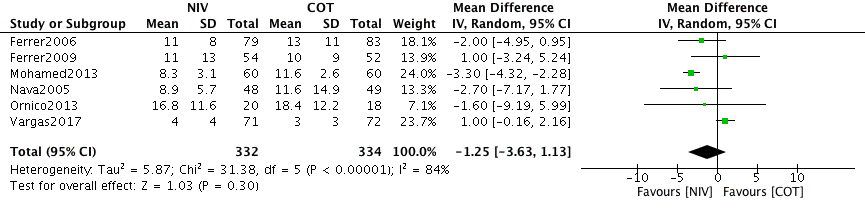


(b) HFNC vs COT


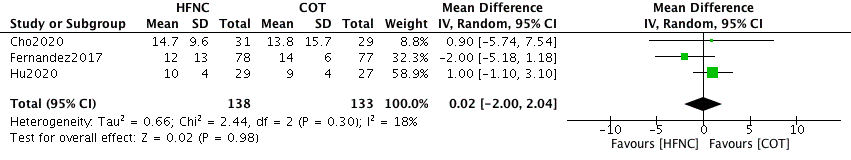


(c) HFNC vs NIV


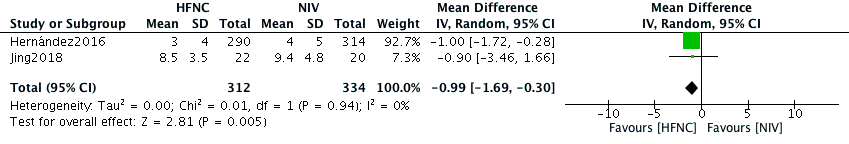


(d) NIV+HFNC vs HFNC


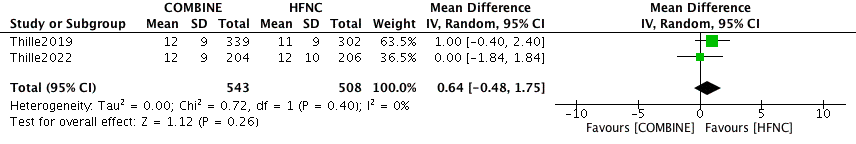


**Figure S4. Forest plots for the pairwise comparison of ICU stay.**

COT conventional oxygen therapy, NIV noninvasive ventilation, HFNC high flow nasal catheter, ICU intensive care unit

(a) NIV vs COT


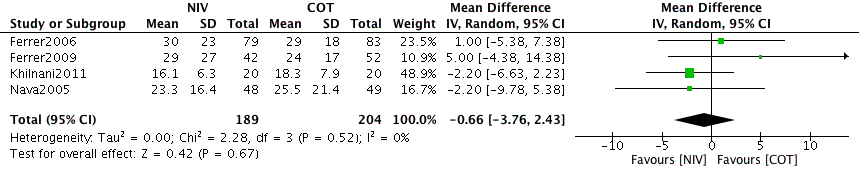


(b) HFNC vs COT


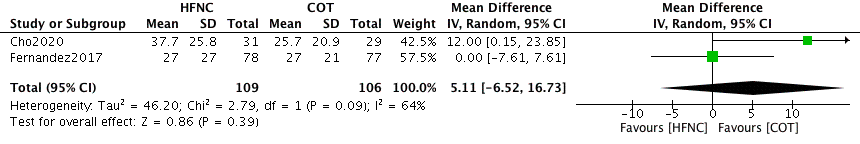


(c) HFNC vs NIV


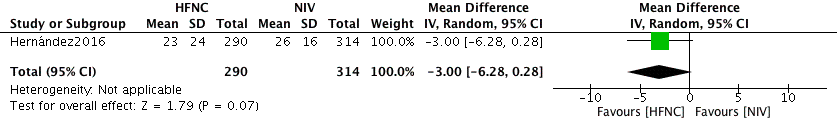


(d) NIV+HFNC vs HFNC


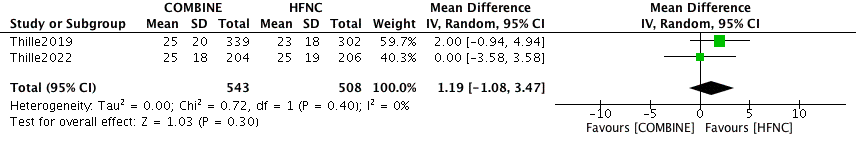


**Figure S5. Forest plots for the pairwise comparison of hospital length of stay.**

COT conventional oxygen therapy, NIV noninvasive ventilation, HFNC high flow nasal catheter

(a)


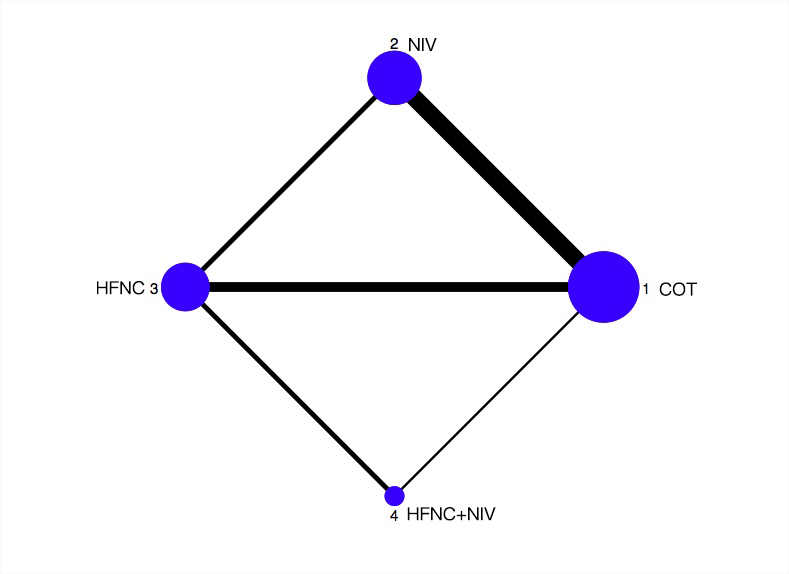


(b)


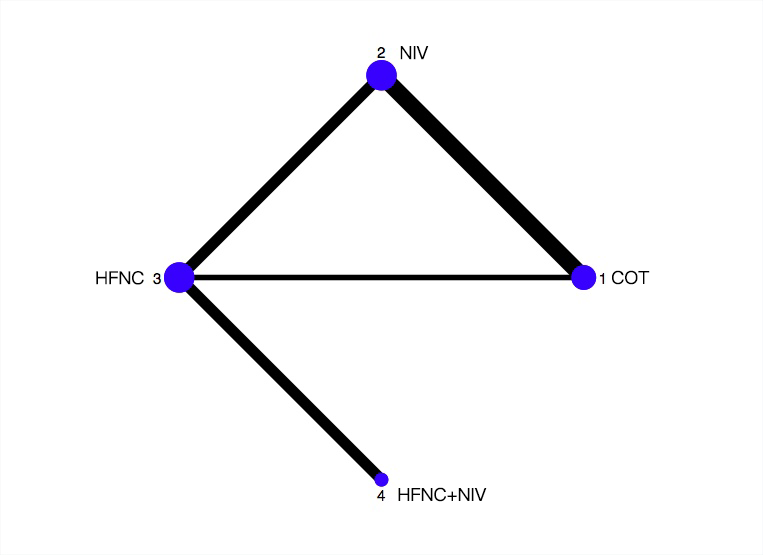


**Figure S6. Network plots correlating respiratory support methods with primary outcomes. (a) reintubation; (b) respiratory failure.**

*COT, conventional oxygen therapy; NIV, noninvasive ventilation; HFNC, high-flow nasal cannula.*


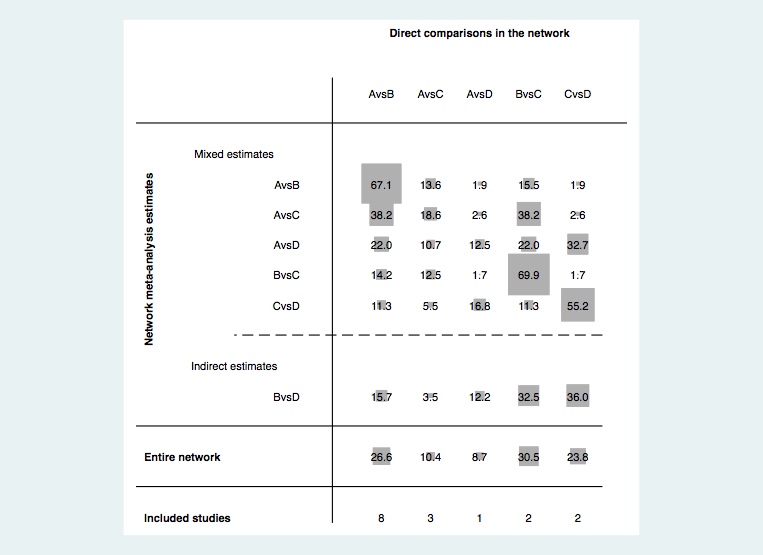


**Figure S7. Weight contribution matrix for reintubation.**

A COT, B NIV, C HFNC, D NIV+HFNC, COT conventional oxygen therapy, NIV noninvasive ventilation, HFNC high flow nasal catheter


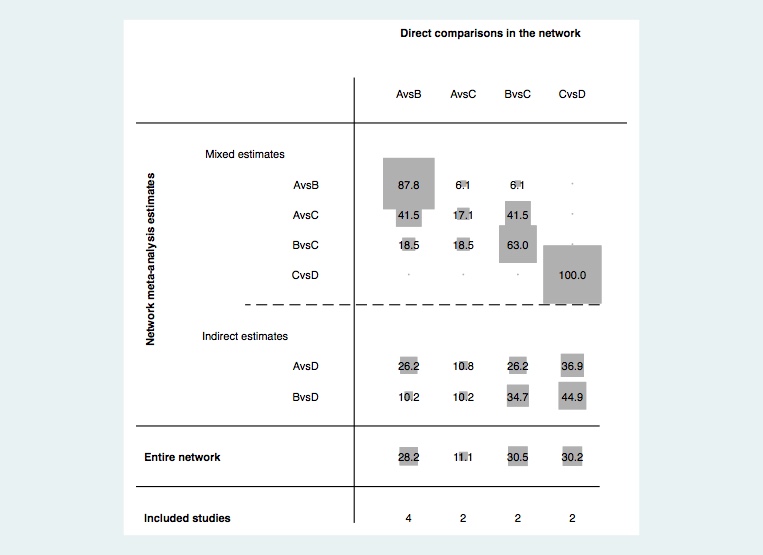


**Figure S8. Weight contribution matrix for post-extubation respiratory failure.**

A COT, B NIV, C HFNC, D NIV+HFNC, COT conventional oxygen therapy, NIV noninvasive ventilation, HFNC high flow nasal catheter


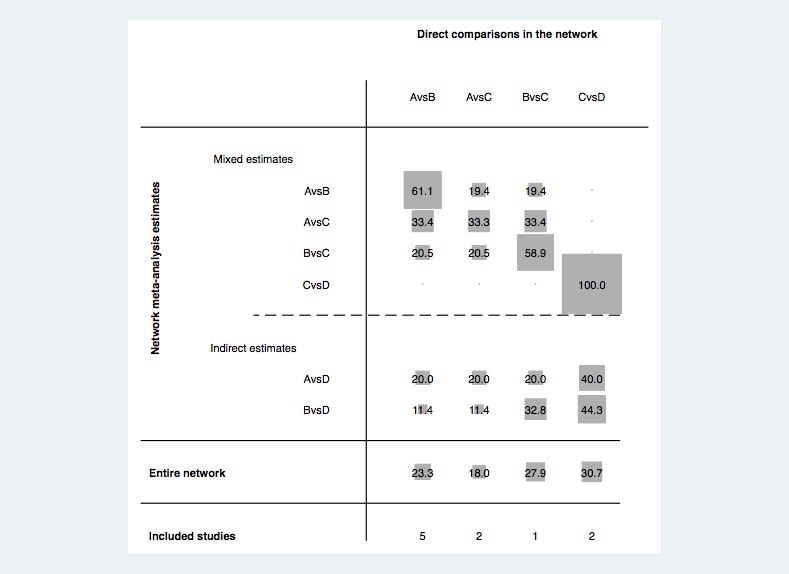


**Figure S9. Weight contribution matrix for ICU mortality.**

A COT, B NIV, C HFNC, D NIV+HFNC, COT conventional oxygen therapy, NIV noninvasive ventilation, HFNC high flow nasal catheter, ICU intensive care unit


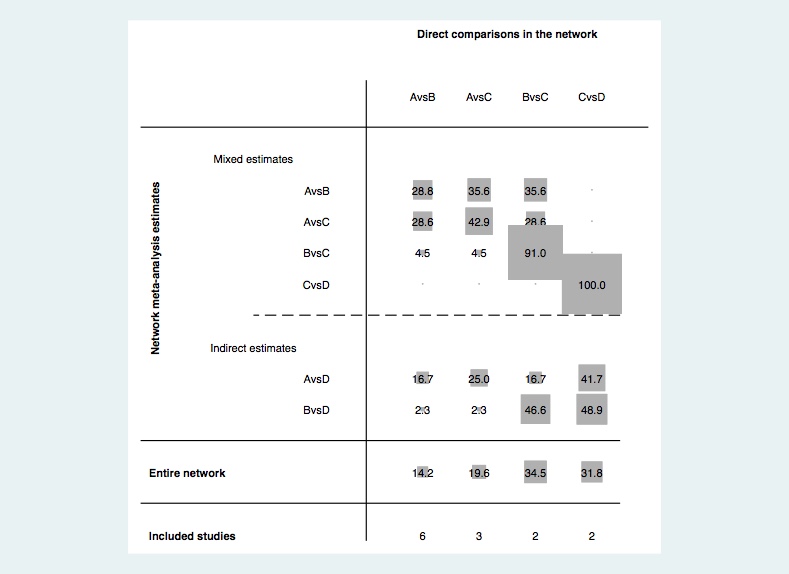


**Figure S10. Weight contribution matrix for ICU stay.**

A COT, B NIV, C HFNC, D NIV+HFNC, COT conventional oxygen therapy, NIV noninvasive ventilation, HFNC high flow nasal catheter, ICU intensive care unit


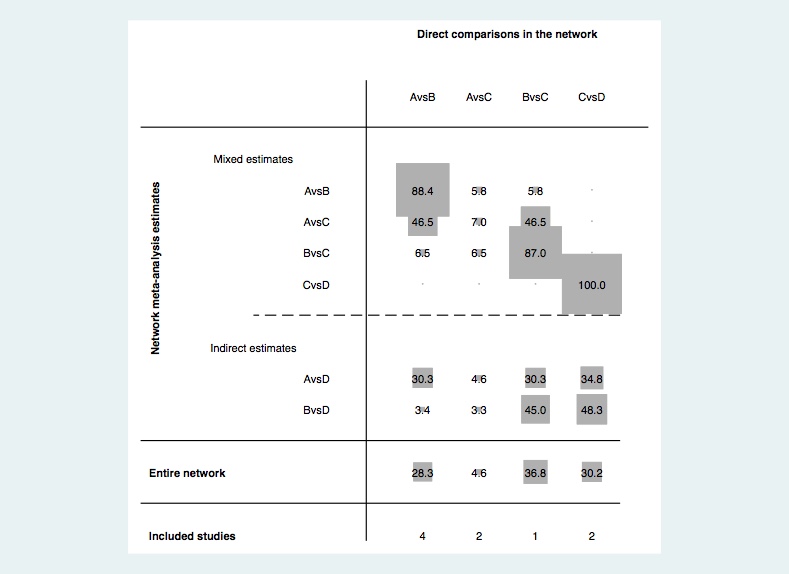


**Figure S11. Weight contribution matrix for hospital length of stay.**

A COT, B NIV, C HFNC, D NIV+HFNC, COT conventional oxygen therapy, NIV noninvasive ventilation, HFNC high flow nasal catheter


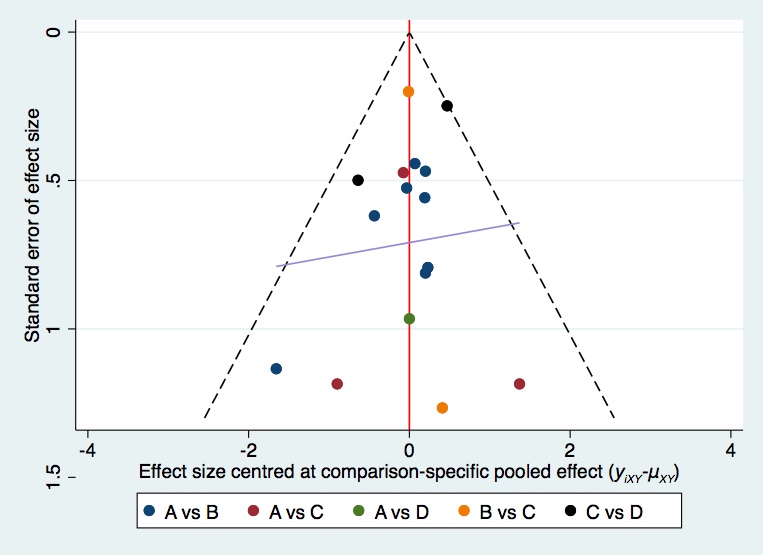


**Figure S12. Funnel plot for reintubation.**

A COT, B NIV, C HFNC, D NIV+HFNC, COT conventional oxygen therapy, NIV noninvasive ventilation, HFNC high flow nasal catheter


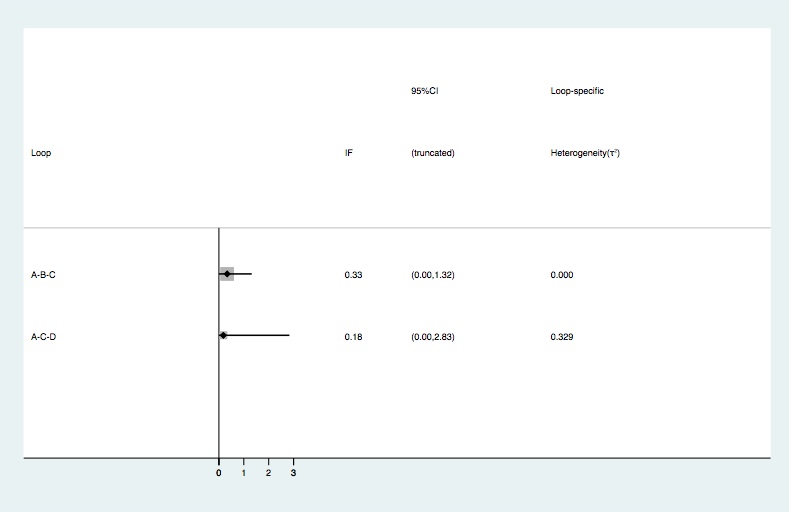


**Figure S13. Inconsistency analysis for reintubation.**

A COT, B NIV, C HFNC, D NIV+HFNC, COT conventional oxygen therapy, NIV noninvasive ventilation, HFNC high flow nasal catheter


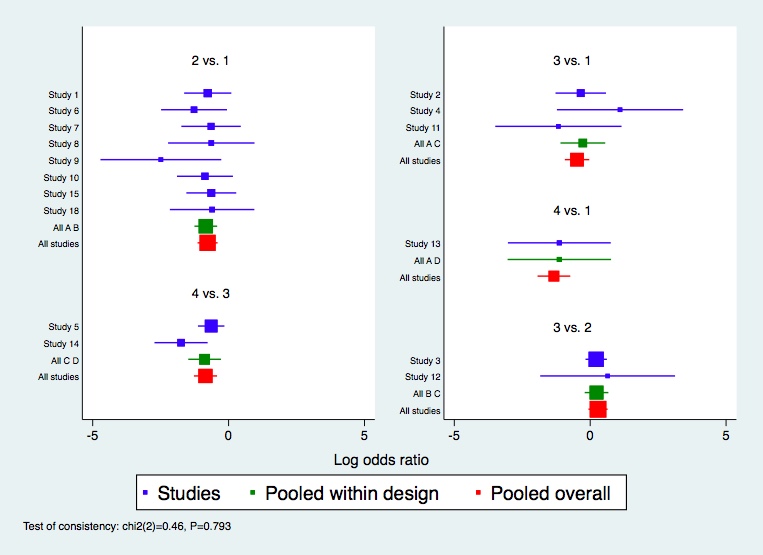


**Figure S14. Forest plot of network meta-analysis for reintubation.**

1 COT, 2 NIV, 3 HFNC, 4 NIV+HFNC, COT conventional oxygen therapy, NIV noninvasive ventilation, HFNC high flow nasal catheter


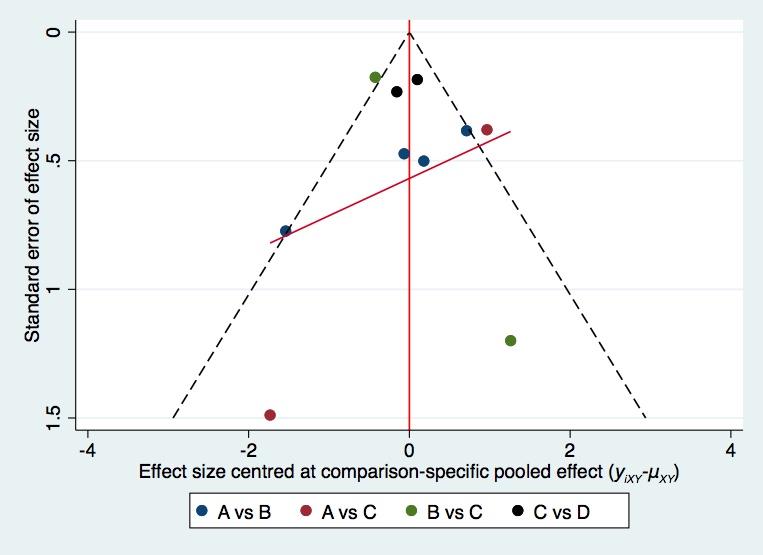


**Figure S15. Funnel plot for respiratory failure.**

A COT, B NIV, C HFNC, D NIV+HFNC, COT conventional oxygen therapy, NIV noninvasive ventilation, HFNC high flow nasal catheter


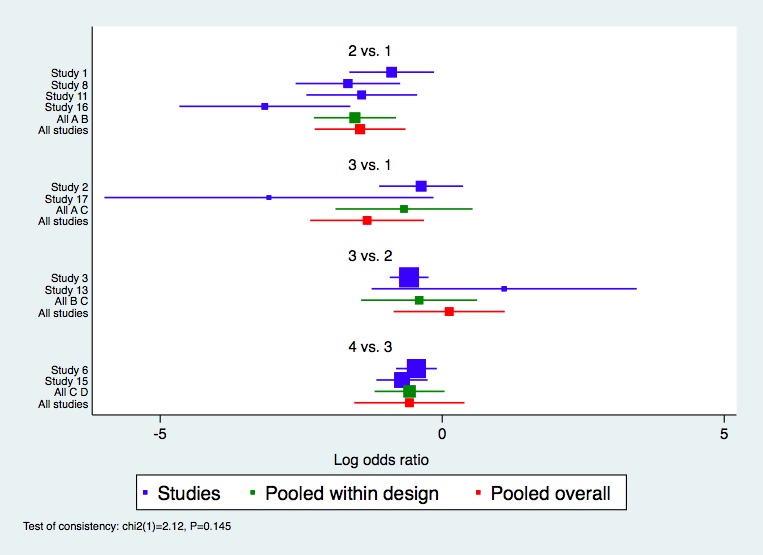


**Figure S16. Forest plot of network meta-analysis for respiratory failure.**

1 COT, 2 NIV, 3 HFNC, 4 NIV+HFNC, COT conventional oxygen therapy, NIV noninvasive ventilation, HFNC high flow nasal catheter


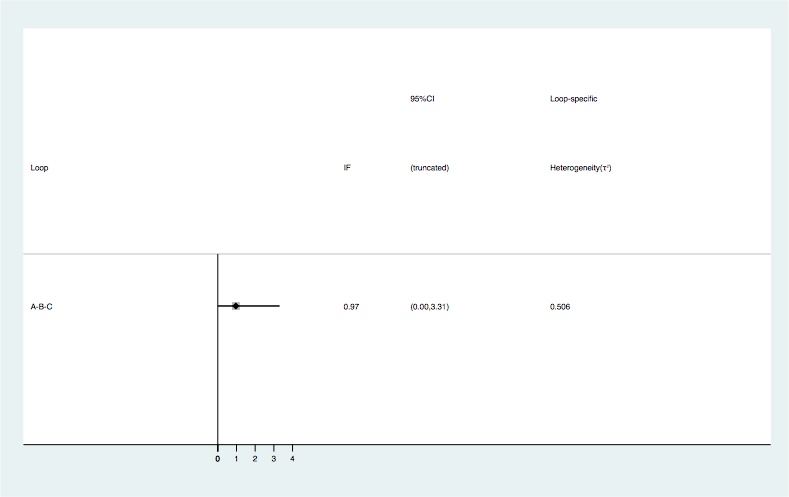


**Figure S17. Inconsistency analysis for respiratory failure.**

A COT, B NIV, C HFNC, COT conventional oxygen therapy, NIV noninvasive ventilation, HFNC high flow nasal catheter


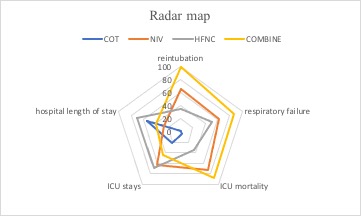


**Figure S18. Radar map of respiratory support methods for reintubation rate, respiratory failure, ICU mortality, ICU stay and LOS.**

*ICU, intensive care unit; LOS, length of stay; NIV, noninvasive ventilation; COT, conventional oxygen therapy; HFNC, high-flow nasal cannula.*


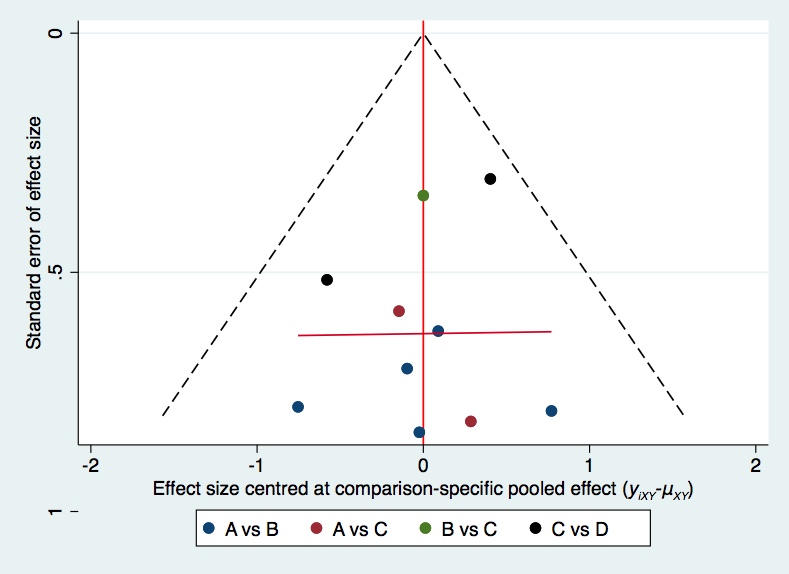


**Figure S19. Funnel plot for ICU mortality.**

A COT, B NIV, C HFNC, D NIV+HFNC, COT conventional oxygen therapy, NIV noninvasive ventilation, HFNC high flow nasal catheter, ICU intensive care unit


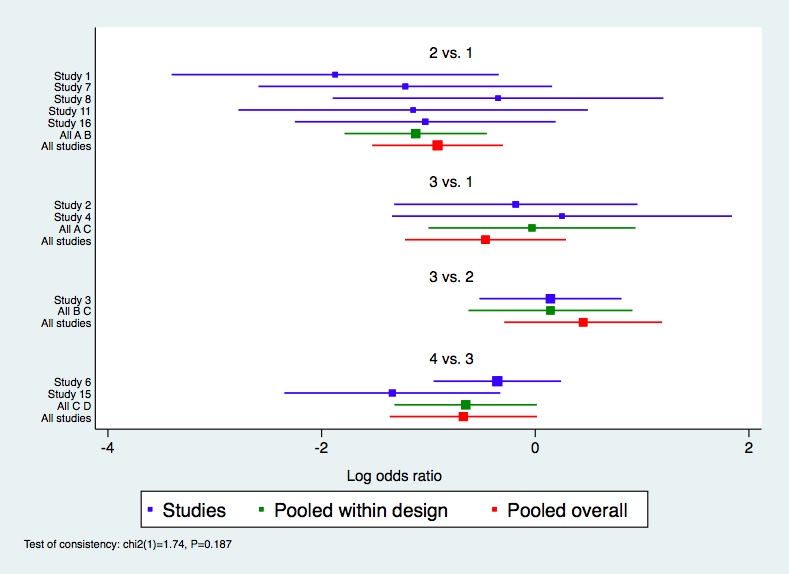


**Figure S20. Forest plot of network meta-analysis for ICU mortality.**

1 COT, 2 NIV, 3 HFNC, 4 NIV+HFNC, COT conventional oxygen therapy, NIV noninvasive ventilation, HFNC high flow nasal catheter, ICU intensive care unit


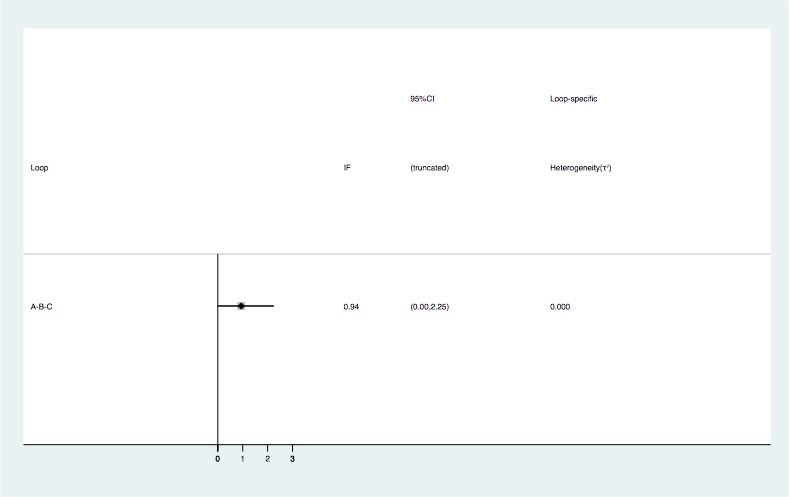


**Figure S21. Inconsistency analysis for ICU mortality.**

A COT, B NIV, C HFNC, COT conventional oxygen therapy, NIV noninvasive ventilation, HFNC high flow nasal catheter, ICU intensive care unit


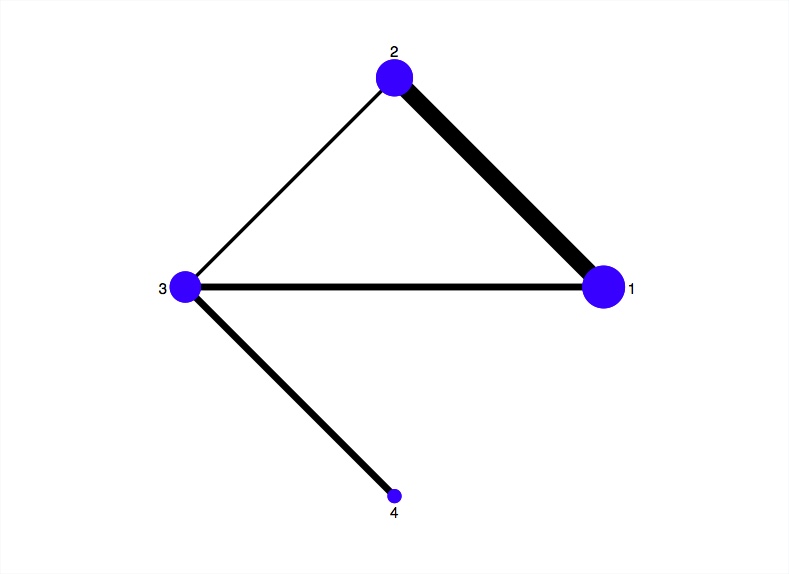


**Figure S22. Network geometry for ICU mortality.**

1 COT, 2 NIV, 3 HFNC, 4 NIV+HFNC, COT conventional oxygen therapy, NIV noninvasive ventilation, HFNC high flow nasal catheter, ICU intensive care unit

The size of the node was proportional to the number of trials that included in each method, and the thickness of the lines was proportional to the number of direct comparisons.


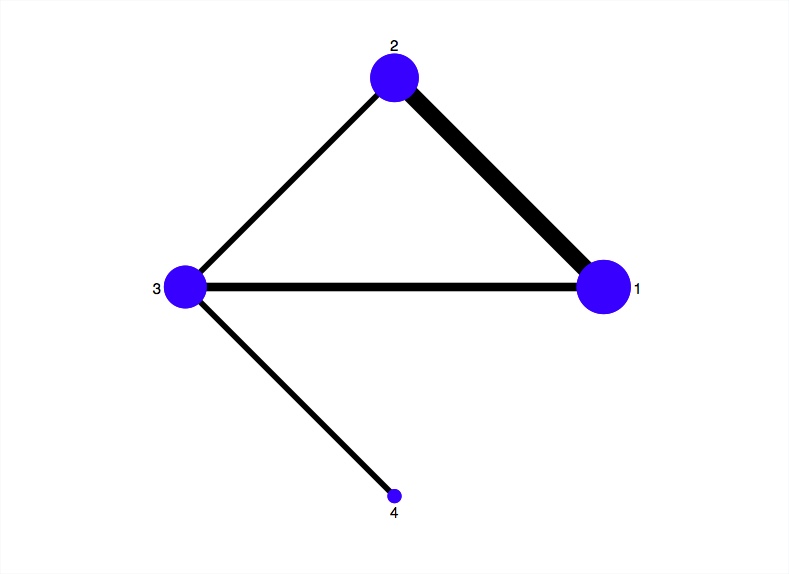


**Figure S23. Network geometry for ICU stay.**

1 COT, 2 NIV, 3 HFNC, 4 NIV+HFNC, COT conventional oxygen therapy, NIV noninvasive ventilation, HFNC high flow nasal catheter, ICU intensive care unit

The size of the node was proportional to the number of trials that included in each method, and the thickness of the lines was proportional to the number of direct comparisons.


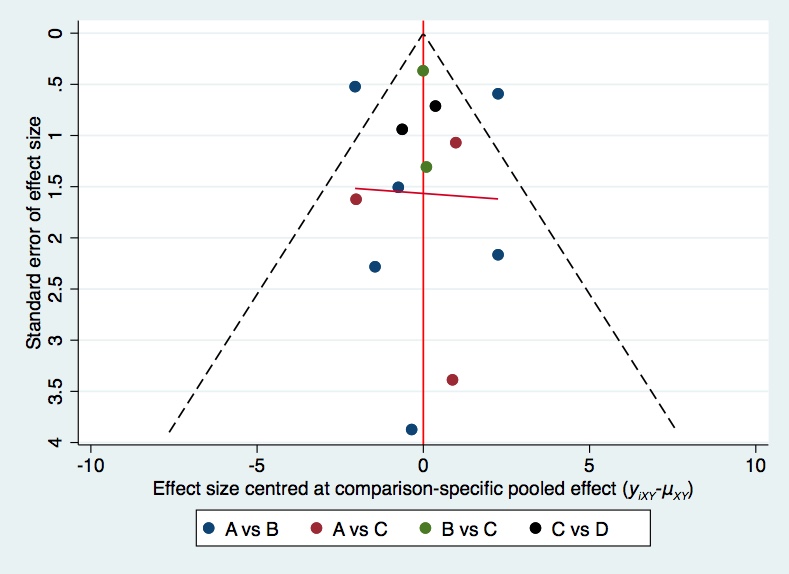


**Figure S24. Funnel plot for ICU stay.**

A COT, B NIV, C HFNC, D NIV+HFNC, COT conventional oxygen therapy, NIV noninvasive ventilation, HFNC high flow nasal catheter, ICU intensive care unit


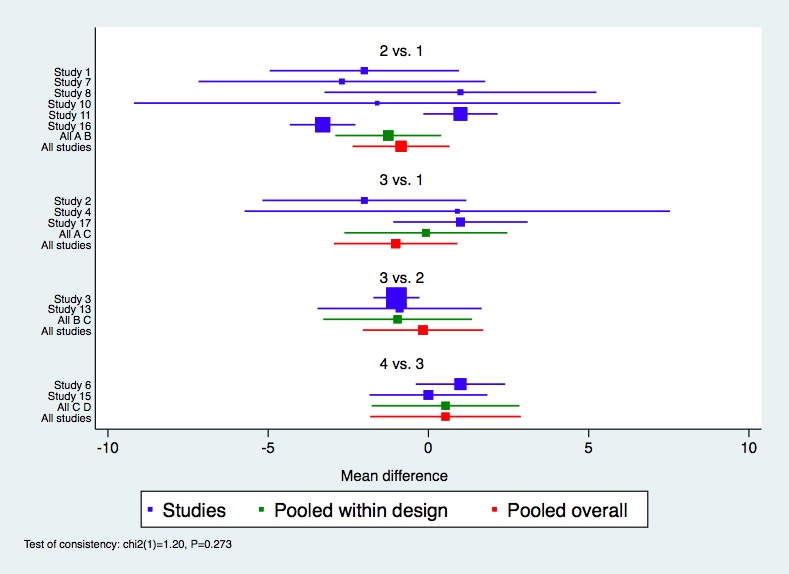


**Figure S25. Forest plot of network meta-analysis for ICU stay.**

1 COT, 2 NIV, 3 HFNC, 4 NIV+HFNC COT conventional oxygen therapy, NIV noninvasive ventilation, HFNC high flow nasal catheter, ICU intensive care unit


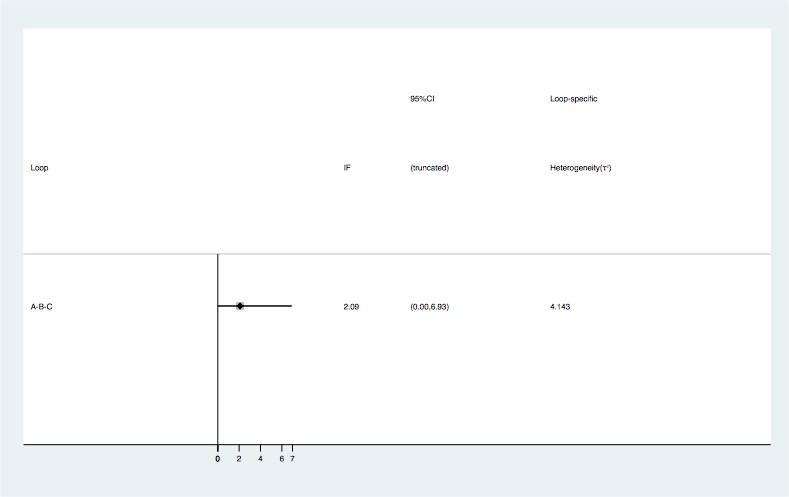


**Figure S26. Inconsistency analysis for ICU stay.**

A COT, B NIV, C HFNC, COT conventional oxygen therapy, NIV noninvasive ventilation, HFNC high flow nasal catheter, ICU intensive care unit


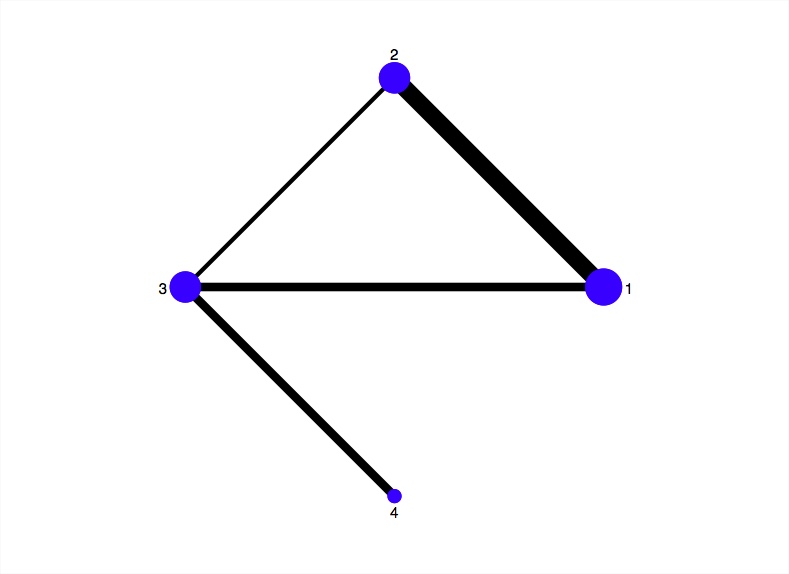


**Figure S27. Network geometry for hospital length of stay.**

1 COT, 2 NIV, 3 HFNC, 4 NIV+HFNC COT conventional oxygen therapy, NIV noninvasive ventilation, HFNC high flow nasal catheter, ICU intensive care unit

The size of the node was proportional to the number of trials that included in each method, and the thickness of the lines was proportional to the number of direct comparisons.


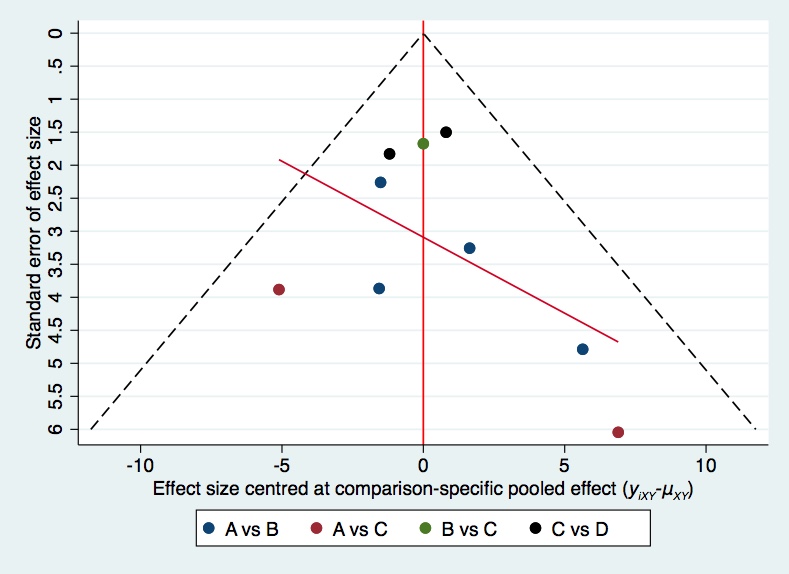


**Figure S28. Funnel plot for hospital length of stay.**

A COT, B NIV, C HFNC, D NIV+HFNC, COT conventional oxygen therapy, NIV noninvasive ventilation, HFNC high flow nasal catheter


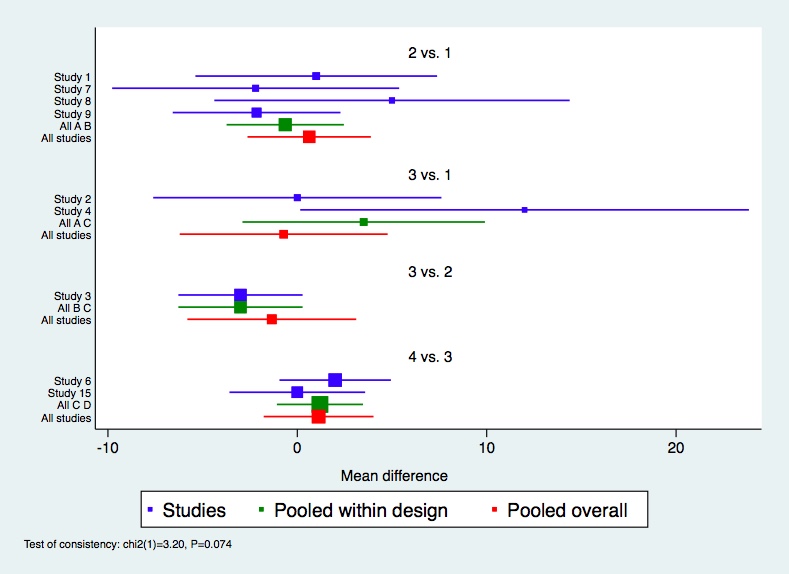


**Figure S29. Forest plot of network meta-analysis for length of stay.**

1 COT, 2 NIV, 3 HFNC, 4 NIV+HFNC, COT conventional oxygen therapy, NIV noninvasive ventilation, HFNC high flow nasal catheter


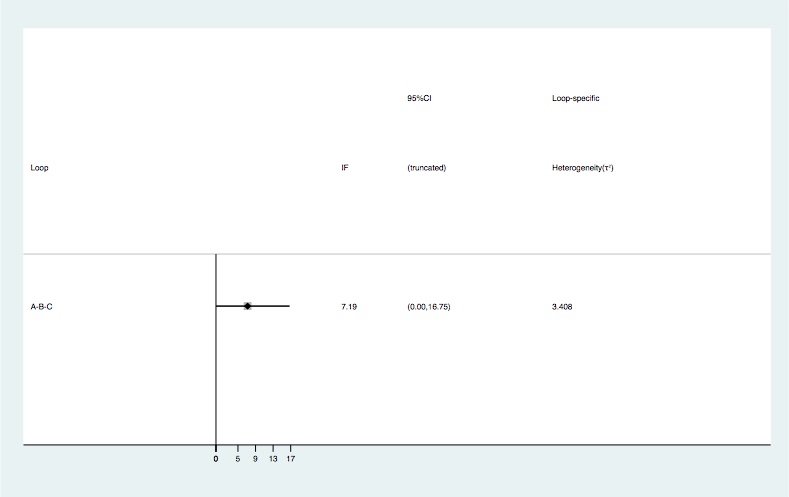


**Figure S30. Inconsistency analysis for length of stay.**

A COT, B NIV, C HFNC, COT conventional oxygen therapy, NIV noninvasive ventilation, HFNC high flow nasal catheter, ICU intensive care unit

(a) reintubation


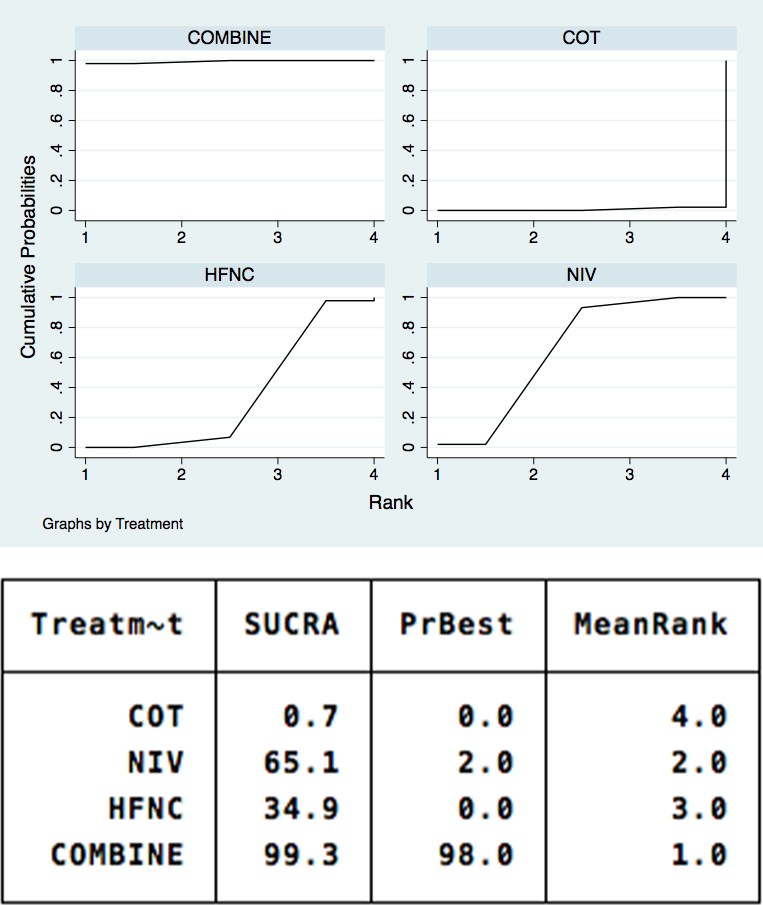


COT conventional oxygen therapy, NIV noninvasive ventilation, HFNC high flow nasal catheter

(b) respiratory failure


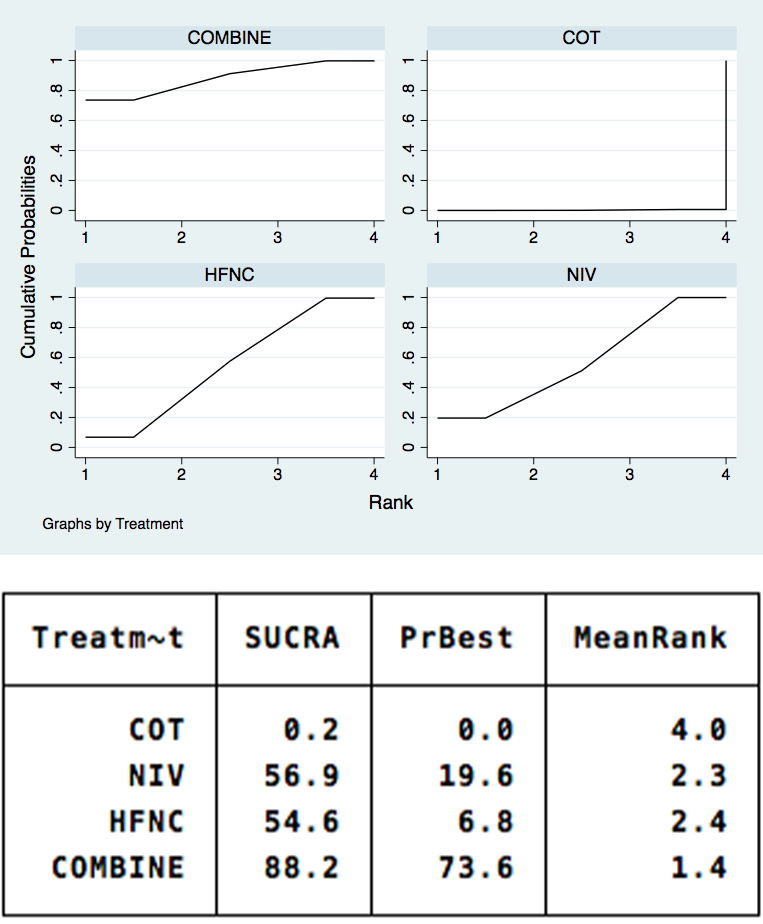


**Figure S31. Surface under cumulative ranking curve of respiratory support methods of sensitivity analysis exclusively including trials with sample size ≥ 50 for the primary outcomes.**

COT conventional oxygen therapy, NIV noninvasive ventilation, HFNC high flow nasal catheter

(a) reintubation


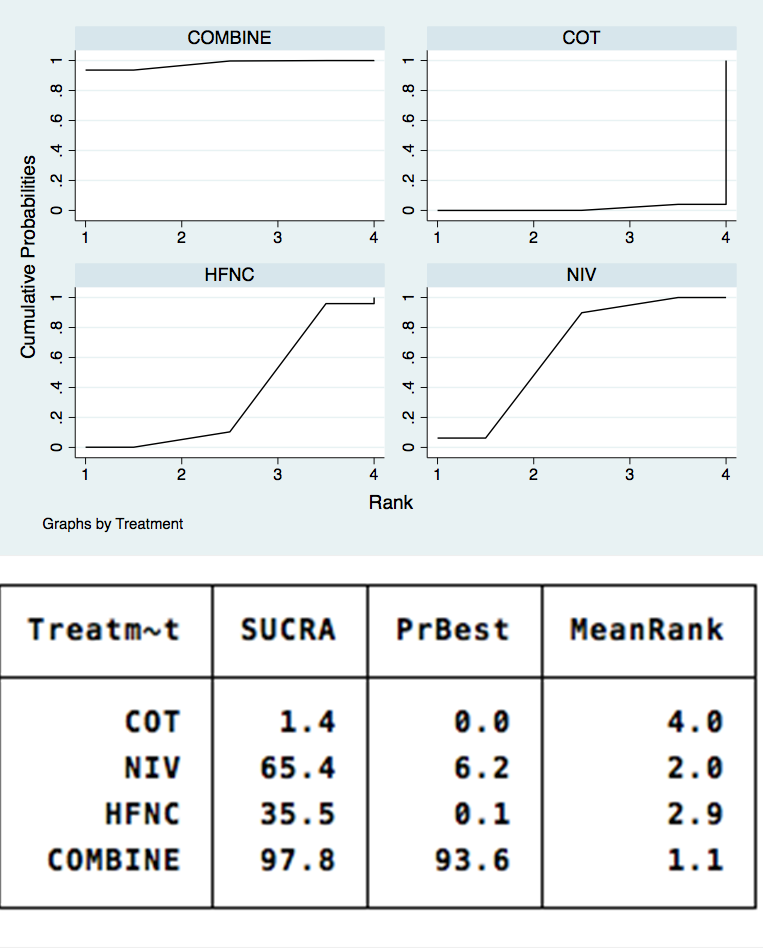


(b) respiratory failure


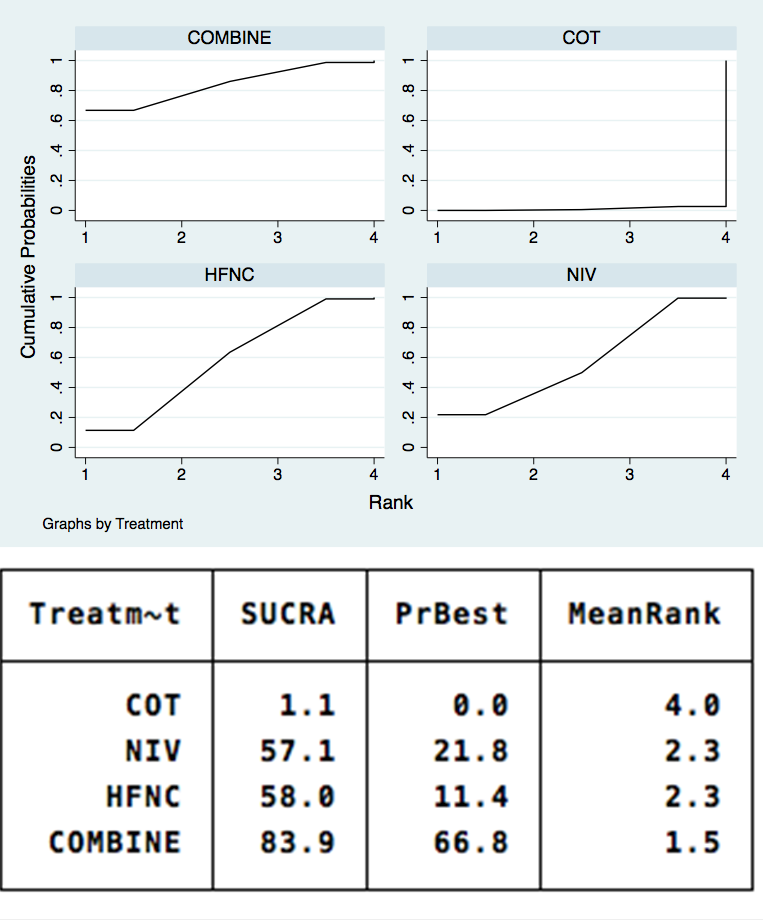


**Figure S32. Surface under cumulative ranking curve of respiratory support methods of sensitivity analysis exclusively including trials with PaCO_2_ ≤ 45 mmHg at the end of SBT for the primary outcomes.**

COT conventional oxygen therapy, NIV noninvasive ventilation, HFNC high flow nasal catheter
